# Extensive Modulation of the Transcription Factor Transcriptome during Somatic Embryogenesis in *Arabidopsis thaliana*

**DOI:** 10.1371/journal.pone.0069261

**Published:** 2013-07-17

**Authors:** Marta Gliwicka, Katarzyna Nowak, Salma Balazadeh, Bernd Mueller-Roeber, Malgorzata D. Gaj

**Affiliations:** 1 Department of Genetics, University of Silesia, Katowice, Poland; 2 Institute of Biochemistry and Biology, University of Potsdam, Potsdam, Germany; 3 Max-Planck Institute of Molecular Plant Physiology, Potsdam-Golm, Germany; University of North Carolina at Charlotte, United States of America

## Abstract

Molecular mechanisms controlling plant totipotency are largely unknown and studies on somatic embryogenesis (SE), the process through which already differentiated cells reverse their developmental program and become embryogenic, provide a unique means for deciphering molecular mechanisms controlling developmental plasticity of somatic cells. Among various factors essential for embryogenic transition of somatic cells transcription factors (TFs), crucial regulators of genetic programs, are believed to play a central role. Herein, we used quantitative real-time polymerase chain reaction (qRT-PCR) to identify TF genes affected during SE induced by *in vitro* culture in *Arabidopsis thaliana.* Expression profiles of 1,880 TFs were evaluated in the highly embryogenic Col-0 accession and the non-embryogenic *tanmei/emb2757* mutant. Our study revealed 729 TFs whose expression changes during the 10-days incubation period of SE; 141 TFs displayed distinct differences in expression patterns in embryogenic versus non-embryogenic cultures. The embryo-induction stage of SE occurring during the first 5 days of culture was associated with a robust and dramatic change of the TF transcriptome characterized by the drastic up-regulation of the expression of a great majority (over 80%) of the TFs active during embryogenic culture. In contrast to SE induction, the advanced stage of embryo formation showed attenuation and stabilization of transcript levels of many TFs. In total, 519 of the SE-modulated TFs were functionally annotated and transcripts related with plant development, phytohormones and stress responses were found to be most abundant. The involvement of selected TFs in SE was verified using T-DNA insertion lines and a significantly reduced embryogenic response was found for the majority of them. This study provides comprehensive data focused on the expression of TF genes during SE and suggests directions for further research on functional genomics of SE.

## Introduction

Most plant cells, in contrast to animal cells, express an amazing developmental plasticity allowing their reprogramming and manifestation of totipotency [1]. Our current understanding of the genetic mechanisms controlling plant totipotency are largely based on studies on somatic embryogenesis (SE), the process through which already differentiated cells reverse their developmental program during *in vitro* culture and become embryogenic giving rise to the formation of somatic embryos which then develop further into entire plants. Thus, deciphering the molecular determinants of SE can directly contribute to revealing the genetic programme underlying the phenomenon of cell totipotency. Moreover, considering similarities between SE and zygotic embryogenesis (ZE), functional genomics of SE became a model for the analysis of the molecular mechanisms of ZE [2], [3]. Importantly, knowledge about the molecular mechanisms governing SE has also a practical value in plant biotechnology for the improvement of existing and the establishment of new protocols for plant regeneration.

The control of plant embryogenesis, similar to other developmental processes, occurs through a complex set of intrinsic signals that are involved in providing information to the dividing and differentiating cells. Of them, phytohormones and transcription factors (TFs) are believed to play central roles [4]. TFs constitute sequence-specific DNA-binding proteins that are capable of activating and/or repressing transcription of target genes and thus are responsible for gene expression regulation. TF genes are often expressed in a tissue- or developmental stage-specific mode or in a stimulus-dependent manner, and many have been shown to obey important roles in developmental processes [5], [6], [7]. Moreover, in adult human somatic cells a specific combination of TFs was found to re-programme differentiated cells into pluripotent embryonic stem cells [8], [9]. More specifically, a combination of only four over-expressed TFs was sufficient to induce the formation of pluripotent stem cells from e.g. adult human fibroblasts [10],[11].

In contrast to the spectacular progress that has been made with respect to the identification of key genetic factors able to transform differentiated animal cells into totipotent stem cells much less is known about the master regulators of genomic reprogramming in plant cells. Of note, transcriptional regulation is thought to play a more important role in plants than in animals and accordingly, recent analyses have recognized over 2,000 TFs to be encoded by the Arabidopsis genome and revealed a higher ratio of TF genes to the total number of genes in this plant than in several animal model organisms such as *Drosophila melanogaster* or *Caenorhabditis elegans* [12].

In agreement with the model that TFs play fundamental roles in the control of plant cell totipotency, genes encoding TFs are currently overrepresented among the genetic factors reported to be essential for SE. The list of genes affecting SE includes *BABY BOOM* (*BBM*) [13], *WUSCHEL* (*WUS*) [14], *AGAMOUS-LIKE15* (*AGL15*) [15], *LEAFY COTYLEDON* (*LEC*) [16], *LEC1*-*LIKE* (*L1L*) [17] and genes encoding MYB transcription factors, i.e., *AtMYB115*, *AtMYB118* [18] and *EMK* (*EMBRYOMAKER*) [19]. Several TFs involved in SE have been reported to enhance plant regeneration efficiency when overexpressed [13], [14], [20].

Various molecular tools have been employed to identify genes essential for embryogenic transition of somatic plant cells. Microarray-based transcriptome analyses were used to discover genes involved in SE induction and somatic embryo development in various plant species including gymnosperms such as *Picea sp.* [21], , cereals such as maize [23] and rice [24], and eudicots, such as e.g. *Glycine max* [25] and *Solanum tuberosum* [26]. In contrast to commonly used DNA microarrays, transcriptome analysis based on quantitative real-time polymerase chain reaction (qRT-PCR) provides an up to 100 times more sensitive tool for transcript detection [27]. With respect to TFs, which are often expressed at a low level or in a cell-specific manner, the superior sensitivity of multi-parallel qRT-PCR over microarray hybridisations has been reported [28]. Recently, multi-parallel qRT-PCR was employed in a number of biological studies, e.g. to determine the expression levels of ∼1,900 TFs in Arabidopsis in response to different carbon sources [29] or phosphorus treatment [30]. Similarly, multi-parallel qRT-PCR has been used to study the expression of more than 2,000 TFs in rice [31], of 1,000 TFs in *Medicago truncatula* [32], or of 1,000 TFs during tomato fruit development [33].

In the present study we took advantage of the available Arabidopsis TF qRT-PCR platform to indentify TF genes involved in the process of SE induced *in vitro* in Arabidopsis cultures. To identify TFs prominently expressed during SE we compared transcriptomes of Arabidopsis genotypes exhibiting largely different embryogenic capacities, namely the highly embryogenic accession Col-0 and the embryonal mutant *tanmei/emb2757* entirely lacking an embryogenic response *in vitro* [34]. Expression of 1,880 TFs was profiled at selected time points during SE culture and TFs prominently expressed in Col-0 were identified. The capacity for SE induction was evaluated in mutants carrying T-DNA insertions in 17 TF genes that showed SE-modulated expression; the majority of the mutants displayed a significantly impaired embryogenic response, indicating that our transcriptome screening indeed revealed genes functionally relevant for SE. Our approach constitutes the first comprehensive analysis of the global TF transcriptome involved in the process of SE induced in plant tissue culture and provides the basis for a better understanding of the genetic determinants of plant developmental plasticity.

## Results

### Experimental Design

To indentify TF genes potentially involved in SE, we employed a well established protocol for the induction of somatic embryos (see Materials and Methods). In brief, immature zygotic embryos (IZEs) at the late cotyledonary stage of development were carefully excised from siliques 10–12 days after pollination and cultured on solid medium containing the synthetic auxin analog 2,4-dichlorophenoxyacetic acid (2,4-D, 5 µM). Induction of SE in this experimental setup is accompanied by distinct morphological changes of the explant. In Arabidopsis (Col-0 accession), a straightening and expansion of previously bent cotyledons and swelling of the cotyledon node are observed during the first week of *in vitro* culture. The first somatic embryos become visible at days 8 to 10, on the adaxial sides of the cotyledons proximal to the cotyledon node, and at around day 15 the cotyledon part of the immature zygotic embryo is covered with somatic embryos at various stages of development [35].

**Figure 1:**
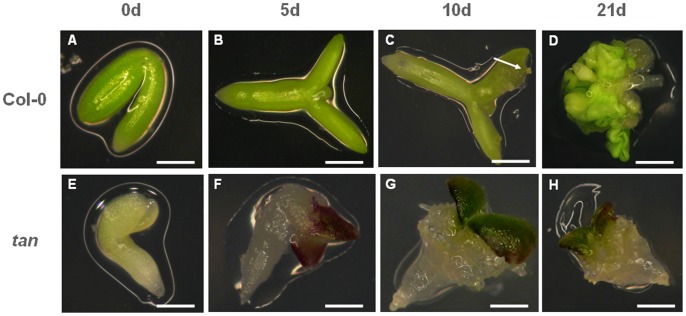
Developmental changes in Arabidopsis Col-0 and *tanmei* IZE explants induced on auxin-containing medium. A–D) Col-0 accession. E–H) *tanmei* mutant. Explants were induced on auxin-containing medium (E5) and monitored at days 0 (A, E), 5 (B, F), 10 (C, G) and 15 (D, H) of *in vitro* culture. A, E) Freshly isolated IZE 12 days after pollination (DAP). B) Straightening, enlargement and swelling of IZE cotyledons. C) Tissue proliferation and somatic embryo-like protuberances formed at adaxial side (arrow). D) Numerous somatic embryos at the adaxial side of IZE cotyledons. F) Anthocyanin accumulation in IZE cotyledons and tissue proliferation from IZE hypocotyl. G) Non-embryogenic watery callus. H) Progression of non-embryogenic callus production. Bars: 0.2 mm (A, B, E, F); 0.3 mm (C, G); 0.6 mm (H) and 1.0 mm (D).

The experiment was designed to monitor the expression of 1,880 TF genes at three distinctive stages of IZE-derived embryogenic culture: (i) freshly isolated explants (0 d), (ii) explants subjected to SE induction for 5 days (5 d), and (iii) explants at an advanced stage of embryogenesis related to somatic embryo formation (10 d). To identify genes exhibiting preferential expression during SE, we compared the TF transcriptomes of the highly embryonic Col-0 accession and the *tanmei* mutant unable to form somatic embryos (**Figure 1**). The *TANMEI*/*EMB2757* (*TAN*, At4g29860) gene encodes a regulatory WD repeat protein involved in early and late phases of zygotic embryo development [36] as well as SE [34]. Its molecular mode of action has not been reported yet, however, the fact that TAN harbours seven WD repeats suggests that it interacts with other proteins to exert its biochemical function. Recently, a regulatory function of TANMEI in cell cycle progression and differentiation was reported [37].

**Figure 2:**
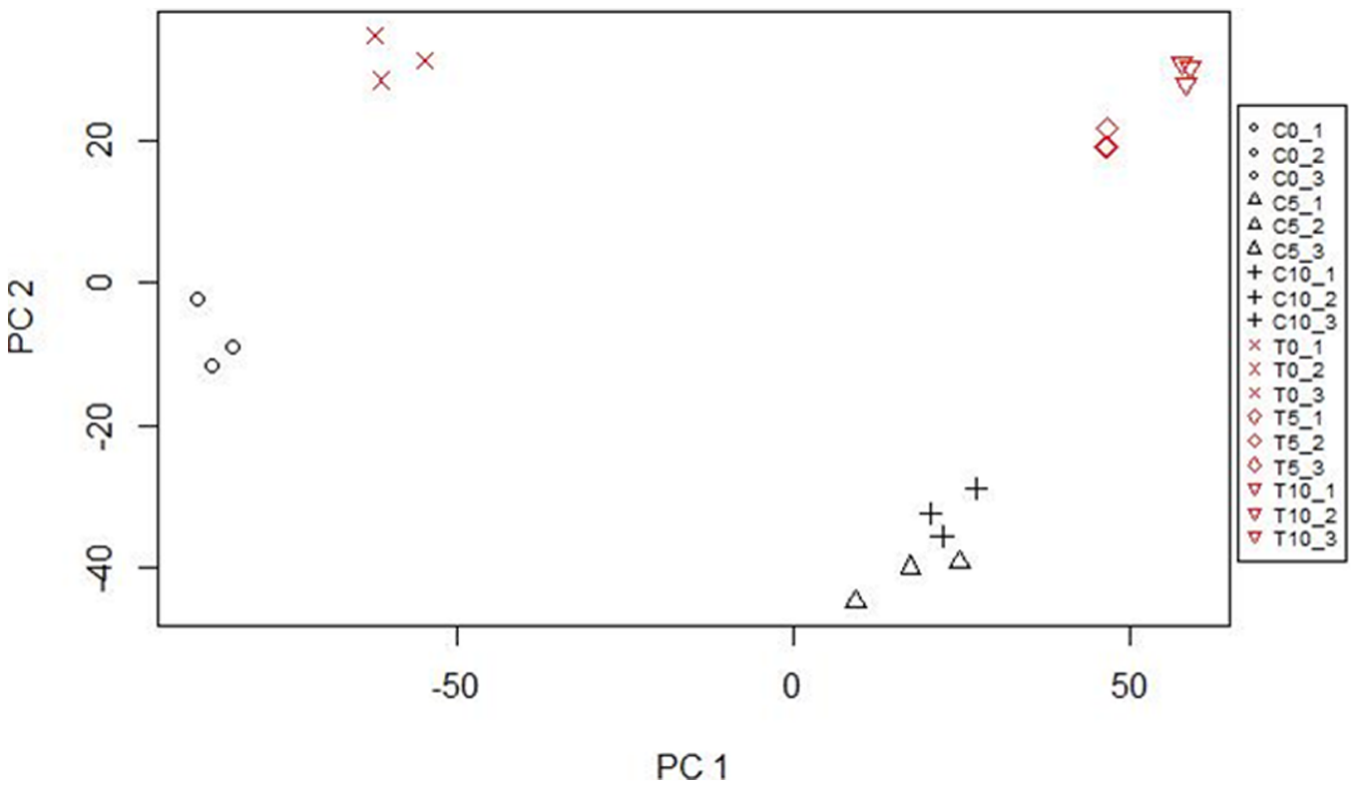
Principal Component Analysis (PCA). The analysis demonstrates a clear separation of TF expression in Col-0 and *tanmei* (*tan1–2*), both in explants (0 d) and during embryogenic culture (5 d and 10 d). Expression data from three independent biological replicates were analysed each. Samples: C0, Col-0, day 0; C5, Col-0, day 5; C10, Col-0, day 10; T0, *tan* mutant, day 0; T5, *tan* mutant, day 5; T10, *tan* mutant, day 10. Numbers 1 to 3 denote replicates 1 to 3. Approximately 67.6% of the variation is captured by the first two components.

PCA (Principal Component Analysis; **Figure 2**) and HCA (Hierarchical Cluster Analysis; not shown) demonstrated high reproducibility of the three experimental replicates performed, i.e., samples representing biological repeats of the same combination (genotype x culture time point) grouped together. In addition, we observed a clear separation of samples from different combinations indicating that expression profiles of embryogenic Col-0 and non-embryogenic *tanmei* tissues differ significantly. Moreover, the 5 d- and 10 d-Col-0 embryogenic cultures tended to overlap indicating similarities between the TF transcriptomes of the different stages of embryogenic culture.

### TF Genes Related to Embryogenic Competency of Explant Tissue

**Figure 3:**
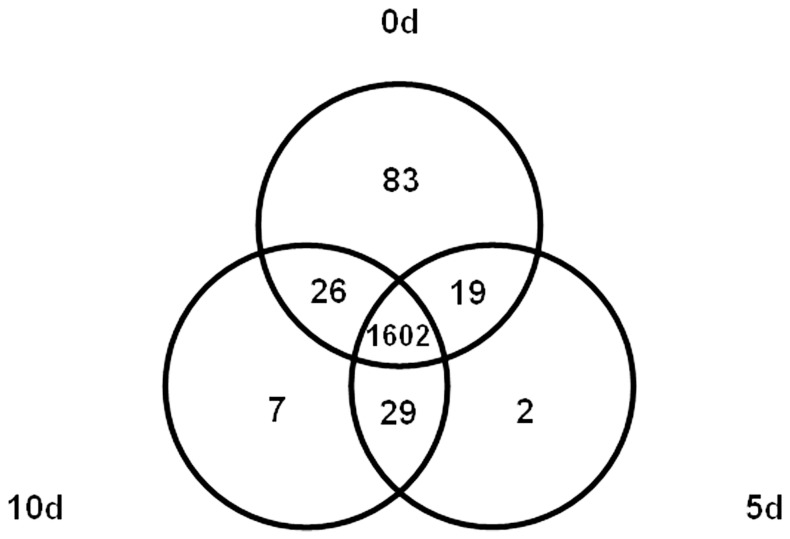
Venn diagram demonstrating the number of genes expressed during SE in the Col-0 accession. Numbers in intersections represent TFs commonly expressed at the different culture time points: 0 d, explant; 5 d, induction phase of SE; 10 d, advanced SE culture.

In Col-0, a large number of TFs were expressed at the different time points (0, 5 and 10 d) of the culture (**Figure 3**). The biggest number of TFs was expressed in explants before embryogenic induction (0 d) and 83 of them were repressed thereafter. Of the TFs analysed, 1602 were expressed in all culture stages, whilst SE stage-specific transcripts were rare and limited to two and seven for the 5-d and 10-d culture time points, respectively.

**Figure 4:**
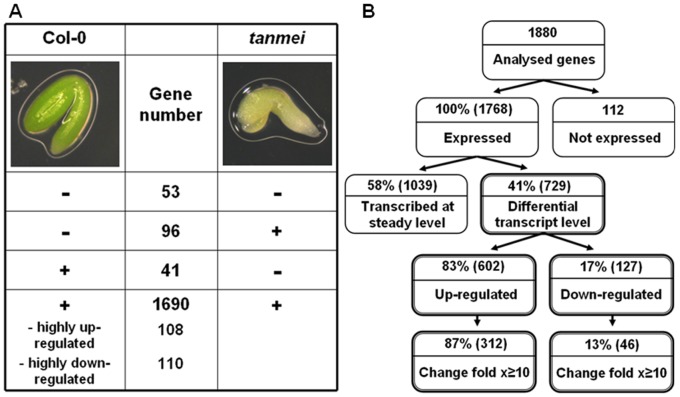
Numbers of TF genes expressed in explants of the highly embryogenic Col-0 genotype and the non-embryogenic *tanmei* mutant (A) and in embryogenic Col-0 culture (B). ´+ ´, genes for which expression was observed; ´- ´, genes for which no expression was observed. Differentially expressed and highly-regulated genes show at least 2- (x≥2) and 10-fold (x≥10) change, respectively, in expression level in any of the compared culture points.

To identify TFs specific for SE-competent tissue we compared the Col-0 and *tanmei* transcriptomes (**Figure 4A**). This revealed expression of 1727 TFs, of which 1690 were commonly expressed in both types of explants. With respect to genes related to embryogenic competency of somatic tissue, transcripts highly enriched in Col-0 versus *tanmei* were of particular interest. Following this criterion, 41 TFs only expressed in Col-0 and TFs highly overexpressed (over 10-fold) in Col-0 versus *tanmei* (108) were inspected further; for 61 TFs a function was predicted, including genes related to stress tolerance, zygotic embryogenesis, developmental processes, hormone biology and *in vitro* responses (**Table S1**). We found that one third (44) of the TFs highly enriched in Col-0 explants were differentially expressed in the embryogenic culture. The set of genes highly up-regulated (at least 10-fold) exclusively in Col-0 explants and SE-modulated in the derived cultures includes TFs related to stress responses (12) and development of zygotic embryos (10), flowers (4), leaves (2) and roots (1).

### The Global TF Transcriptome changes during Somatic Embryogenesis in Col-0

Of the 1,880 TFs analysed, 1,768 were found to be expressed in Col-0 explants in at least one of the three time points, and only 112 TFs were not expressed at any stage (**Figure 4B**). To gain insight into TF expression patterns associated with SE we compared the expression levels observed in explant tissue (0 d) to the expression levels obtained after 5 d (early embryo induction) and 10 d (advanced embryo formation) of culture.

**Figure 5:**
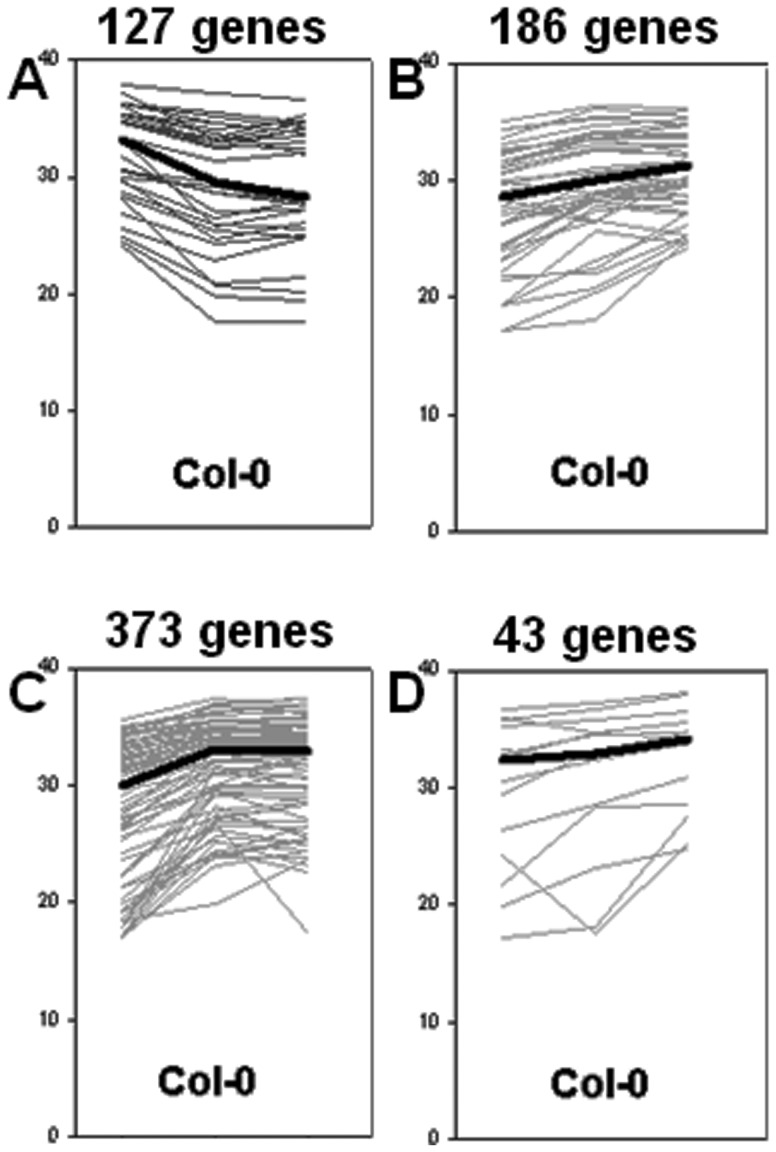
Cluster analysis. *K*-means clustering revealed four main expression patterns of TF genes in Col-0 embryogenic cultures. The levels of expression changes are given as 40-ddCt. The cluster analysis shows up-regulation of the great majority of TFs (B, C, D), and down-regulation of a small group of TFs (A). Increased TF expression was either restricted to the early stage of SE (C), or was observed during both SE stages, early and advanced (B, D).

Our analysis revealed 729 TFs (representing ∼41% of all detected TFs) to be differentially expressed (by at least 2-fold) in embryogenic cultures versus explants (**Figure 5B** **; Table S2**). A closer inspection of the transcriptomes associated with embryo induction identified 673 and 688 genes, respectively, that were modulated at early (5 d vs. 0 d) and advanced (10 d vs. 0 d) stages of SE. The vast majority (602 TFs; 83%) of the modulated TFs were up-regulated, rather than down-regulated, compared to the initial explant (0 d) transcriptome. Of the TFs modulated during SE, 358 displayed a dramatic change in expression level (x≥10) and most (312 TFs; ∼87%) were found to be up-regulated.

The transcript levels detected in the 0-, 5- and 10-d samples were subjected to *k*-means clustering and four major gene expression patterns were observed (**Figure 5**). The cluster analysis confirmed that most TFs were up-regulated in embryogenic cultures; the increased expression was either dominant during the early stages of SE induction (**Figure 5C**), or was observed during both SE stages, early and advanced (**Figure 5B,D**).

In summary, global transcriptome analysis identified an extensive expressional reprogramming of TF genes during SE, where an up-regulation of TF expression was predominantly observed.

### TF Transcriptomes of Early (Embryo-induction) versus Advanced (Embryo-formation) SE Stages

Given that the early days of an embryogenic culture are critical for embryogenic transition of somatic tissue and decisive for the transcriptional re-programming of the explant, we focused our further analysis on TFs undergoing expression changes during the early embryogenic response. To reveal TFs modulated during SE induction, we compared the transcriptome of the 5-d culture with that of the explant (5 d–0 d) and the 10-d embryo culture (5 d–10 d) (**Table 1**). Our analysis revealed that TF transcriptomes associated with the early and advanced SE stages differed significantly with respect to the level and direction of the expression changes. In contrast to SE induction (5 d vs. 0 d), ∼2.5 times fewer genes (284 vs. 673) were differentially expressed between the early and late embryo formation stages (10 d vs. 5 d) and a number of up-regulated genes was distinctly decreased resulting in a similar fraction of up- (154) and down- (130) regulated TFs in the advanced, embryo-formation culture stage. In addition, at the embryo formation stage (10 d vs. 5 d) differentially expressed genes exhibited less drastic changes in transcription and accordingly, the number of genes (32) exhibiting an at least 10-fold change in expression between the 5 d- and 10 d-cultures was over 10 times lower than in a preceding SE induction stage (5 d vs. 0 d).

**Table 1:** Number of TF genes whose expression changes during somatic embryo formation.

Compared culture stages	Number of genes showing differential expression	Up-regulated genes	Down-regulated genes
**Fold change x≥2**			
5 d–0 d	673	546 (81%)	127 (19%)
10 d–0 d	688	542 (79%)	146 (21%)
10 d–5 d	284	154 (60%)	130 (40%)
**Fold change x≥10**			
5 d–0 d	357	312 (87%)	46 (13%)
10 d–0 d	379	331 (87%)	48 (13%)
10 d–5 d	32	6 (19%)	26 (81%)

x, fold change.

To identify genes modulated at the early culture period, we tracked transcript levels of individual genes during the two successive culture periods (5 d–0 d and 5 d–10 d). To this end, TFs up- or down-regulated, or remaining unchanged during SE induction, were grouped together according to their expression profiles during the subsequent embryo formation stage (**Table 2**). Scrutiny of the individual gene expression patterns revealed that most TFs (67%) up-regulated during embryo induction (5 d) did not significantly change expression thereafter during embryo formation; only few genes were down- (∼15%) or up-regulated (∼18%) in the 10-d culture compared to the 5-d culture.

**Table 2:** Number of differentially expressed TF genes exhibiting convergent expression profiles across SE culture.

Embryo-induction stage (5 d vs. 0 d)^a^		Embryo-forming stage (10 d vs. 5 d)^b^				
Expression change	Number of genes	Down-regulation		Up-regulation		Steady expression
		x≥2	x≥10	x≥2	x≥10	x<2
**Up-regulation**	**x≥2∶546**	81	5	100	11	368
	**x≥10∶307**	62	5	58	3	187
**Down-regulation**	**x≥2∶125**	25	1	40	12	60
	**x≥10∶45**	1	1	25	9	19
**Steady expression**	**x<2∶38**	25	1	13	2	0

x, fold change of gene expression.

a Expression behavior of TF genes within the first five days of somatic embryogenesis.

b Expression change of the genes grouped in column 1 (“Embryo-induction stage”) during the second phase of somatic embryogenesis (expression at day 10 compared with expression at day 5).

Stabilization of the TF transcriptome in advanced cultures was also observed for genes down-regulated during SE induction (5 d–0 d). We found that almost half of the genes (48%) down-regulated during embryo induction were not further modulated at the later stage of embryo formation, whilst the remaining genes were up- (32%) or further down-regulated (20%). In contrast to the vast number of genes differentially regulated during SE induction, a small set of 38 TFs was found to be modulated exclusively in the advanced SE culture. The transcript levels of these genes remained stable until the embryo formation stage when most of them (∼66%) were found to be down-regulated.

To identify TFs specific for SE induction we searched for those that drastically (by at least 10-fold) changed their expression levels during the early culture stages. We identified genes of high and temporal changes in expression specific to SE induction and among them were the key regulators of embryogenic transition induced in cultured cells in response to auxin treatment (**Table S3**).

Collectively, by analyzing TF gene expression profiles across the time points of SE we obtained the following results: (i) The embryo-induction stage of SE is associated with a robust change of the TF transcriptome. (ii) Transcriptome reprogramming during SE induction includes a drastic up-regulation of a great majority (over 80%) of the TFs active in culture. (iii) TF expression patterns of embryo induction and embryo formation stages are largely different. (iv) In contrast to SE induction, attenuation and stabilization of transcript levels of a great fraction of the TFs is observed in the advanced embryo formation stage.

### Col-0 versus *tanmei* Transcriptome and SE-associated Genes

**Figure 6:**
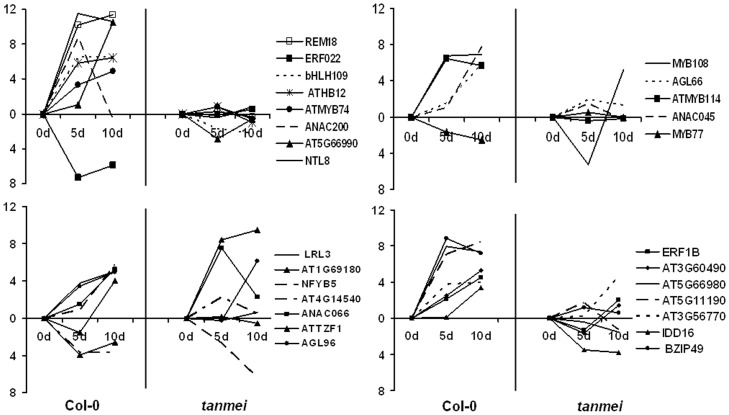
Expression profiles of SE-associated genes. The graph shows contrasting expression levels of TFs in embryogenic (Col-0) and non-embryogenic (*tanmei*) cultures. The relative transcripts levels of the genes are shown as ddCt.

**Figure 7:**
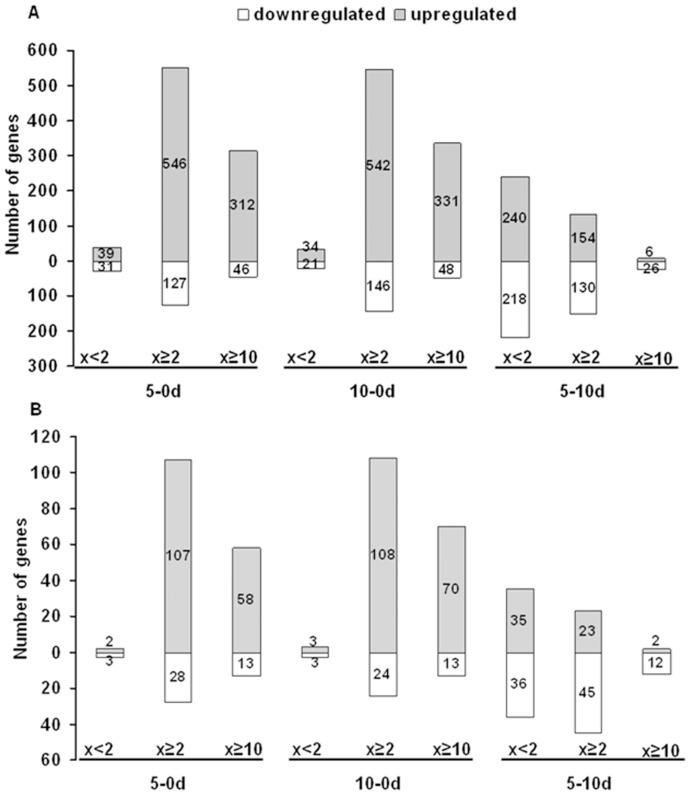
Number of TF genes of modulated expression in embryogenic cultures. A) TFs expressed in Col-0 culture. B) TFs of SE-specific expression pattern, i.e. those displaying distinctly different expression profiles in Col-0 and *tanmei* cultures. Numbers of TFs of steady (fold change<2) and modulated (fold change≥2) expression in embryogenic cultures referenced to the indicated culture time points (i.e. 5 d–0 d; 10 d–0 d, and 5 d–10 d) are given. Genes with up- and down-regulated expression are indicated.

To identify candidate TFs of SE-associated functions we compared the transcriptomes of cultures derived from the highly embryogenic Col-0 genotype and the *tanmei* mutant lacking the embryogenic response; genes of distinctly different expression profiles were selected. We identified 141 TF genes with SE-specific expression (**Table 3**) falling into the following groups: (i) genes exclusively expressed in embryogenic culture (2 genes); (ii) genes differentially expressed in Col-0, but steadily expressed in *tanmei* (72 genes); (iii) genes exhibiting opposite expression patterns in Col-0 and mutant cultures, including genes up-regulated in Col-0 and down-regulated in *tanmei* (33), and genes down-regulated in Col-0 and up-regulated in *tanmei* (10); examples are shown in **Figure** **6**; and (iv) genes significantly down-regulated in non-embryogenic *tanmei* culture (24). We found that, similar to the global Col-0 transcriptome, SE-specific transcripts were predominantly up-regulated during SE and for a substantial part of them the changes in expression level were drastic (x≥10) (**Figure 7**).

**Table 3:** TF genes showing SE-specific expression.

				Fold change 2^ΔΔCt^			
AGI	Gene name	TF family	Known or predicted function	Col-0		*tan*	
				5d–0d	5d–10d	5d–0d	5d–10d
*AT1G02030*		C2H2	Seed germination	30.06	2.10	Steady expression	
*AT1G06170*	*bHLH89/EN24*	bHLH	Flower development, ZE [49]	7.89	4.59	Steady expression	
*AT1G19790*	*SRS7*	SRS	Flower development	3.92	1.32	Steady expression	
*AT1G25250*	*IDD16*	C2H2		1.10	−2.50	Steady expression	
*AT1G25560*	*EDF1/TEM1*	AP2/EREBP	Flowering time	2.95	−9.51	Steady expression	
*AT1G28160*	*ERF087*	AP2/EREBP	Stress	9.51	−1.88	Steady expression	
*AT1G34650*	*HDG10*	HB		25.63	−14.72	Steady expression	
*AT1G44830*		AP2/EREBP	Biotic stress	6.19	−2.79	Steady expression	
*AT1G51220*		C2H2		2.50	1.21	Steady expression	
*AT1G54330*	*ANAC020*	NAC		16.34	−2.64	Steady expression	
*AT1G59640*	*BIG PETAL*	bHLH	Flower development	4.47	−1.29	Steady expression	
*AT1G59810*	*AGL50*	MADS	Flower development	32.67	−1.12	Steady expression	
*AT1G60920*	*AGL55*	MADS		340.14	3.41	207.94	−1.47
*AT1G65300*		MADS	Seed/embryo development	64.45	1.66	3.51	−1.56
*AT1G66380*	*MYB114*	MYB	ZE [49], cell wall	88.65	1.32	Steady expression	
*AT1G67030*	*ZFP6*	C2H2	Cell cycle	89.26	1.78	13.36	−2.03
*AT1G68240*		bHLH		92.41	−2.00	Steady expression	
*AT1G68480*		C2H2	Flower development	3.51	−1.01	Steady expression	
*AT1G77850*	*ARF17*	ARF	Auxin	3.84	−1.78	2.48	1.56
*AT1G77980*	*AGL66*	MADS	Flower development	2.93	−1.47	Steady expression	
*AT2G17150*		NIN-like		4.53	−19.43	Steady expression	
*AT2G23740*	*SUVR5/SET6*	C2H2	Flower development	5.86	−1.45	2.17	−1.28
*AT2G27300*	*ANAC040/NTL8*	NAC	Salt stress	2957.17	2.38	Steady expression	
*AT2G30590*	*WRKY21*	WRKY	SE *Dactilis glomerata* [121]	5.78	1.95	Steady expression	
*AT2G31650*		SET-domain	Histone methylation	4.99	1.55	3.41	−1.80
*AT2G33480*	*ANAC041*	NAC		2.73	1.78	Steady expression	
*AT2G35700*		AP2/EREBP	Biotic stress	2.19	−2.35	1.88	1.46
*AT2G38470*	*WRKY33*	WRKY	Biotic and abiotic stress	4.11	−3.20	−1.07	−1.92
*AT2G39880*	*MYB25*	MYB		5.39	−1.99	3.41	1.09
*AT2G42280*		bHLH		4.38	−1.01	Steady expression	
*AT2G44430*		MYB	Flower development	4.86	−1.32	Steady expression	
*AT2G46770*	*EMB2301/NST1*	NAC	ZE [49], cell wall	67.65	−1.06	44.32	2.64
*AT2G47810*	*NFYB5*	CCAAT-HAP3	Flower development	1.88	−2.20	Steady expression	
*AT2G47890*	*COL13*	C2C2(Zn) CO-like	Flower development	4.92	−29.04	Steady expression	
*AT3G01220*	*ATHB20*	HB	Auxin	9.13	3.01	Steady expression	
*AT3G03200*	*ANAC045*	NAC		2.04	−2.14	Steady expression	
*AT3G04730*	*IAA16*	Aux/IAA	Auxin	16.34	−105.42	Steady expression	
*AT3G06490*	*MYB108/BOS1*	MYB	JA, GA, stress	107.63	−1.34	Steady expression	
*AT3G10470*		C2H2	Flower development	625.99	−1.13	55.33	1.35
*AT3G17600*	*IAA31*	Aux/IAA	ZE [38]	7.62	3.43	Steady expression	
*AT3G17730*	*ANAC057*	NAC		12.64	1.08	Steady expression	
*AT3G19070*		GARP-G2-like	Cell wall	22.01	1.01	Steady expression	
*AT3G21890*	*MZN24.1*	C2C2(Zn) CO-like	Light	15.03	−2.22	Steady expression	
*AT3G23240*	*ERF1B*	AP2/EREBP	Ethylene	4.53	4.23	Steady expression	
*AT3G24310*	*MYB71*	MYB		195.36	−5.13	Steady expression	
*AT3G27940*	*LBD26*	AS2 (LOB) I		128.89	−7.41	199.47	−5.31
*AT3G30260*	*AGL79*	MADS	Root development	14.32	−2.60	6.63	−5.24
*AT3G50700*	*ATIDD2*	C2H2		9.13	−9.00	Steady expression	
*AT3G51080*	*GATA9*	C2C2(Zn) GATA	ZE	3.63	−1.10	Steady expression	
*AT3G53200*		MYB		103.25	−1.41	6.63	−30.48
*AT3G56660*	*BZIP49*	bZIP	Stress	467.88	−1.65	Steady expression	
*AT3G56770*		bHLH	Biotic stress	13.55	3.18	Steady expression	
*AT3G60490*		AP2/EREBP	Stress	5.78	−1.16	−3.18	−8.46
*AT3G61890*	*ATHB-12*	HB	Water and salt stress	56.10	−6.68	Steady expression	
*AT3G61910*	*ANAC066/NST2*	HB	Cell wall	2.81	−1.57	Steady expression	
*AT4G00940*		C2C2(Zn) DOF		8.22	−13.74	Steady expression	
*AT4G01260*		GeBP		55.72	1.80	Steady expression	
*AT4G01540*	*NTM1/ANAC068*	NAC	Cell cycle; cytokinins	6.68	1.32	Steady expression	
*AT4G05100*	*MYB74*	MYB	JA; ethylene; stress	9.92	−1.09	Steady expression	
*AT4G17460*	*HAT1*	HB		24.93	−2.99	Steady expression	
*AT4G20970*		bHLH		64.89	−1.20	765.36	−1.22
*AT4G22070*	*WRKY31*	WRKY	SE *Solanum tuberosum* [26]	2225.63	5.74	Steady expression	
*AT4G22680*	*MYB85*	MYB	Vascular tissue, cell wall	124.50	6.32	Steady expression	
*AT4G24540*	*AGL24*	MADS	Flowering time	4.76	5.35	1.48	−1.12
*AT4G27950*	*CRF4*	AP2/EREBP	Ethylene, stress	10.93	−1.57	Steady expression	
*AT4G28110*	*MYB41*	MYB	ABA, water and salt stress	6.02	−1.93	Steady expression	
*AT4G28500*	*ANAC073/SND2*	NAC		116.97	−1.02	Steady expression	
*AT4G30080*	*ARF16*	ARF	ZE	6.23	1.16	4.17	−1.93
*AT4G32280*	*IAA29*	Aux/IAA	Auxin; root development	94.35	1.52	Steady expression	
*AT4G32730*	*MYB3R1*	MYB	Cell cycle; cytokinins	5.54	1.05	Steady expression	
*AT4G38620*	*MYB4*	MYB	ZE [49]	11.55	1.23	2.43	1.11
*AT4G38910*	*ATBPC5*	BPC/BRR		7.52	5.66	34.78	1.85
*AT4G39250*	*ATRL1*	MYB-related	Seed/embryo development	115.36	−1.47	167.73	35.26
*AT4G39410*	*WRKY13*	WRKY		4.17	11.31	1.73	−1.84
*AT5G01200*		MYB-related		41.07	−3.18	Steady expression	
*AT5G02350*		CHP-rich	Root development	5.03	1.04	−1.15	4.00
*AT5G04390*		C2H2		60.55	4.11	10.85	−1.29
*AT5G06500*	*AGL96*	MADS	ZE [49]	10.63	1.65	Steady expression	
*AT5G06510*	*NF-YA10*	CCAAT-HAP2	Seed/embryo development	24.59	−2.99	17.51	4.14
*AT5G06650*	*GIS2*	C2H2	GA	6.23	−2.22	Steady expression	
*AT5G10030*	*OBF4*	bZIP	ABA, SA, biotic stress	24.42	2.50	Steady expression	
*AT5G11190*		AP2/EREBP	Ethylene, biotic stress	136.24	−1.15	Steady expression	
*AT5G14000*	*ANAC084*	NAC	ZE [49]	8.51	−2.55	Steady expression	
*AT5G15130*	*WRKY72*	WRKY	ZE [49]	1652.00	−1.21	229.13	1.19
*AT5G18000*		B3	Flower development	1184.45	1.93	Steady expression	
*AT5G22890*		C2H2	Root development	94.35	−2.25	Steady expression	
*AT5G23260*	*AGL32/TT16*	MADS	Seed/embryo development	20.53	3.14	Steady expression	
*AT5G24110*	*WRKY30*	WRKY		11746.96	−5.46	Steady expression	
*AT5G26870*	*AGL26*	MADS	Root development	2.08	3.56	Steady expression	
*AT5G26950*	*AGL93*	MADS		11.16	−2.73	Steady expression	
*AT5G27070*	*AGL53*	MADS		18.64	−1.09	Steady expression	
*AT5G27130*	*AGL39*	MADS	Seed/embryo development	10.13	−1.57	1067.48	1.93
*AT5G27580*	*AGL89*	MADS		14.22	2.50	Steady expression	
*AT5G27910*	*NF-YC8*	CCAAT-HAP5		8.46	−1.46	1.26	−2.93
*AT5G38800*	*ATbZIP*	bZIP	Epidermal developmental, cell wall	243.88	−1.31	Steady expression	
*AT5G39760*		ZF-HD		5.82	1.06	4.29	−2.16
*AT5G40220*	*AGL43*	MADS		80.45	2.17	Steady expression	
*AT5G43175*		bHLH		1120.56	−1.11	9741.98	1.58
*AT5G50570*		SBP		5.66	1.03	Steady expression	
*AT5G50670*		SBP		4.86	−1.12	Steady expression	
*AT5G51780*		bHLH		5.66	−2.64	Steady expression	
*AT5G52260*	*MYB19*	MYB		44.63	1.67	Steady expression	
*AT5G56200*	*DEL1/E2L3*	C2H2	Endoreduplication	103.97	1.83	1.21	−47.84
*AT5G58010*	*LRL3*	bHLH	Root development	13.64	−2.43	Steady expression	
*AT5G60440*	*AGL62*	MADS	Seed/embryo development	5.46	1.47	Steady expression	
*AT5G62165*	*AGL42*	MADS		55.72	−15.56	103.25	−1.33
*AT5G66870*	*ASL1/LBD36*	AS2 (LOB) I	Flower development	12.55	1.11	3.18	2.45
*AT5G66980*		B3	Flower development	250.73	1.55	Steady expression	
*AT5G66990*		NIN-like		2.04	−709.18	2.85	−41.36
*AT2G17150*		NIN-like		4.53	−1.45	Steady expression	
*AT2G23740*	*SUVR5/SET6*	C2H2	Flower development; histone	5.86	2.38	2.17	−1.28
			methylation				
*AT1G33760*	*ERF022*	AP2/EREBP	Ethylene, stress	−155.42	−2.60	Steady expression	
*AT1G43640*	*TLP 5*	TUB	Protein degradation	−6.32	1.36	−14.32	−33.36
*AT1G49190*	*ARR19*	GARP-ARR-B	ZE [38]	−25.28	1.27	Steady expression	
*AT1G77200*	*ERF037*	AP2/EREBP	Callus differentiation *O. sativa* [43]	−3.48	2.11	Steady expression	
			ZE globular stage [49]				
*AT2G25900*	*ATTZF1*	C3H	ZE [49]	−15.35	−2.57	Steady expression	
*AT2G42150*		MYB	Seed/embryo development	−4.00	1.60	Steady expression	
*AT3G02310*	*AGL4/SEP2*	MADS	Flower development	−7.89	−3.12	−3.10	−1.24
*AT3G02940*	*MYB107*	MYB	ZE [38]	−65.80	−14.83	Steady expression	
*AT3G03760*	*LBD20/ASL21*	AS2 (LOB) I		−6.68	−4.11	Steady expression	
*AT3G27810*	*MYB21*	MYB	JA, GA	−162.02	1.38	Steady expression	
*AT3G50060*	*MYB77*	MYB	ZE [45], auxin response,lateral root growth	−3.20	1.83	Steady expression	
*AT3G57600*	*DREB2F/ERF051*	AP2/EREBP	Water stress	−1.84	2.64	−2.77	7.84
*AT4G01250*		WRKY	Biotic stress	−3.25	3.39	Steady expression	
*AT4G14540*		CCAAT-HAP3		−12.64	0.00	4.82	3.51
*AT4G32800*		AP2/EREBP	Stress	−4.17	1.21	Steady expression	
*AT4G36900*	*DEAR4/RAP2.10*	AP2/EREBP	Root development; biotic stress	−12.38	−1.45	−1.89	−1.48
*AT4G38000*	*DOF4.7*	C2C2(Zn) DOF	Flower development	−7.67	1.58	Steady expression	
*AT5G04400*	*ANAC077*	NAC		−89.88	1.02	Steady expression	
*AT5G15800*	*AGL2/SEP1*	MADS	Flower development	−34.30	−2.75	Steady expression	
*AT5G39660*	*DOF5.2*	C2C2(Zn) DOF	Flowering time, root development	−4.72	−3.51	Steady expression	
*AT5G51990*	*DREB1D/CBF4*	AP2/EREBP	Water stress	−121.10	−3.51	−110.66	1.05
*AT5G65100*		EIL	Flower development	−3.12	8.51	−71.51	−2.27
*AT5G65590*		C2C2(Zn) DOF		−25.99	−1.13	Steady expression	
*AT5G27810*		MADS		−15.56	−1.42	Steady expression	
*AT5G43840*	*HSFA6A*	HSF	Heat stress	−6.19	−1.69	Steady expression	
*AT4G28790*		bHLH		−1.99	5.17	−1.95	1.21
*AT1G69180*		YABBY	Flower development	−2.97	−48.84	344.89	−2.03
*AT2G14210*	*AGL44/ANR1*	MADS	ZE [49]	−2.64	−52.71	Steady expression	
*AT3G46770*	*REM22*	B3	Flower development	−106.15	−213.78	Steady expression	
*AT4G00870*		bHLH	Flowering time	−1.10	−7.16	3.20	−1.93
*AT4G25480*	*DREB1A/CBF3*	AP2/EREBP	ABA, water stress	−3.61	−13.55	Steady expression	
*AT1G19040*		NAC		424.61	639.15	Steady expression	

### Annotation of Differentially Expressed Genes

The TF genes differentially expressed in embryogenic Col-0 culture were annotated to 50 gene families of which 14 included the great majority (541 genes; 74%) of the differentially expressed transcripts (**Figure S1**). The most frequently represented families were bHLH (75), AP2/EREBP (69), MYB (62), NAC (54), C2H2 (49); WRKY (45), HB (41) and MADS (38), each of which represents 5–11% of the SE-modulated genes.

We next analysed the representation of TF families within the set of SE-associated genes. The SE-associated genes represented 32 TF families and particularly enriched were the MADS (20), MYB (16), AP2/EREBP (15), C2H2 (12), NAC (11), bHLH (11) and ABI3/VPI (4) families. We also noticed that several SE-associated genes belong to the WRKY (7) and DREB (7) families known for their involvement in stress responses.

### Functional Categories of Differentially Regulated Genes

**Figure 8:**
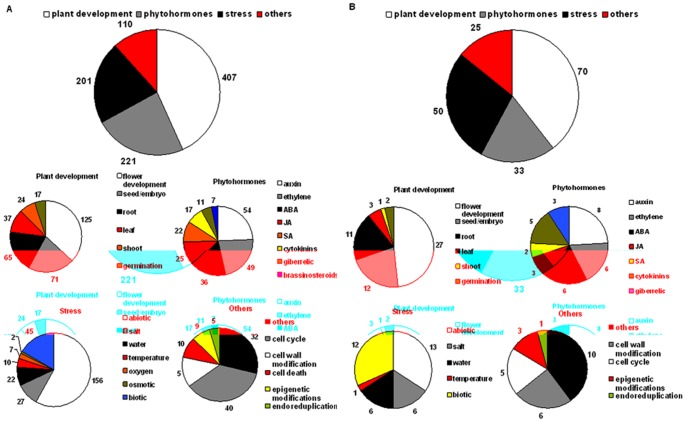
Functional categories of differentially expressed genes. A) TFs differentially expressed during SE. B) SE-associated TFs. TFs were annotated to four major categories (plant development, phytohormones, stress and others) and various subcategories. Given are the numbers of TFs in the different functional categories.

To provide an overview of the potential contribution of TF genes to the regulatory mechanisms involved in SE, the genes differentially expressed in embryogenic culture were annotated according to their known or predicted functions (**Figure 8A**). In total, 519 genes (71%) were functionally annotated and the analysis revealed that the most abundant transcripts are related to plant development, phytohormone biology and stress responses. A great majority (∼78%, 407) of the SE-modulated TFs are related to plant development and in particular TFs involved in flower development were highly abundant (24%; 125). Other numerously represented genes of the plant development category were reported to be involved in embryo and seed development (∼22%, 71).

**Figure 9:**
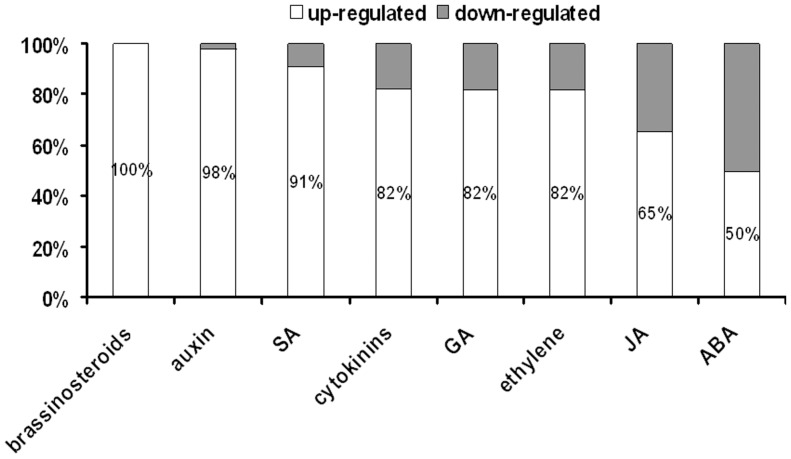
Hormone-related TFs. The graph shows the percentages of hormone-related TFs up- or downregulated in embryogenic Col-0 culture. A great majority of the hormone-related TFs is up-regulated including those related to brassinosteroids, auxin, SA, cytokinins, GA, ethylene, JA and ABA.

The number of TFs related to phytohormones and stress responses were similar and these functional categories included ∼43% and ∼39% of the genes, respectively. Within 221 hormone-related, SE-modulated TFs all major classes of phytohormones were represented and the most numerous were genes related to auxin (∼24%, 54). Half of the auxin-related genes encoded major auxin signaling molecules: ARF (7) and AUX/IAA (20). Beside auxin-related TFs, many genes related to other phytohormones (e.g. ethylene, ABA, cytokinin, GA) were observed to be prevalently up-regulated during SE (**Figure 9**). Among 201 stress-related TFs modulated during SE, genes responding to different abiotic stress factors (salt, water, temperature, oxidative stress) were represented more frequently than those involved in pathogen responses.

Within the group of functionally annotated SE-modulated TFs, 101 (∼20%) represented SE-specific expression, and the number and representation of functional categories were similar to those of globally affected genes (**Figure 8B**). A great majority (∼70%) of the SE-specific TFs were annotated to plant developmental processes and predominantly contribute to flower development (∼27%).

We observed some notable differences between SE-modulated and SE-associated genes with respect to functional annotations. Strikingly, the number of stress-responsive TFs, especially those related to biotic stress, was higher (∼50%) among SE-associated transcripts, whilst the percentage of phytohormone-related genes was lower (∼33%), but interestingly the representation of cytokinin- and gibberellin-related genes was increased over auxin-related genes.

### Functional Test of SE-modulated Transcription Factors

**Figure 10:**
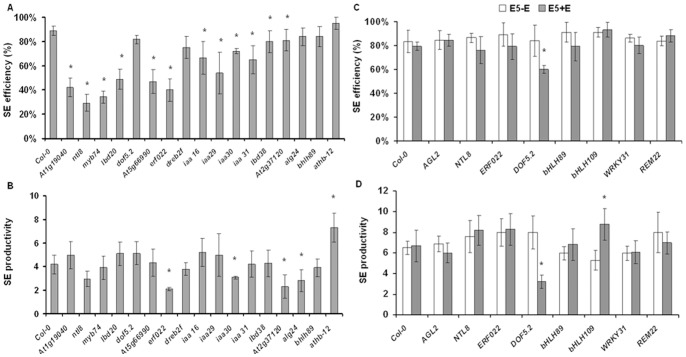
Functional test of SE-modulated transcription factors. Embryogenic capacity of TF T-DNA insertion mutants (A, B) and transgenic lines expressing the indicated TFs under the control of a ß-estradiol-inducible promoter (C, D) was analysed and SE efficiency (A, C) and SE productivity (B, D) were evaluated. Values significantly different from the parental Col-0 genotype are marked by asterisks (n  = 3; means ± SD are given; Mann-Whitney’s U test; p<0.05).

To further elucidate the involvement of TFs in SE we analysed the capacity for SE induction in mutants carrying T-DNA insertions in 17 TF genes of SE-modulated expression (**Table S4**). Twelve of them (∼70%) were found to display a significantly impaired embryogenic response manifested by a reduced number of explants undergoing embryogenic transition (**Figure 10A**). The SE-defective phenotypes suggest that the mutated TFs contribute to SE induction; however, the precise molecular functions of most of the genes are unknown. Among the mutants showing reduced embryogenic potential were those affected in genes related to auxin signaling (*AUX/IAA*). All *iaa* mutants analysed (i.e., *iaa16, iaa29, iaa30* and *iaa31*) displayed significantly impaired SE efficiency, manifested by a lower frequency of explants undergoing SE induction compared to the Col-0 wild type (**Figure 10A**). Furthermore, one of them (*iaa30*) also produced significantly fewer somatic embryos per responding explant (**Figure 10B**).

In addition to the analysis of the insertion mutants, the capacity for SE was evaluated in eight transgenic lines overexpressing TFs of SE-modulated expression under the control of a ß-estradiol-inducible promoter (**Figure 10C,D**). We observed a significantly reduced embryogenic response in cultures overexpressing *DOF5.2*; both, SE efficiency and SE productivity were impaired, i.e. fewer explants underwent SE induction and a lower number of somatic embryos were produced by the responding explants, indicating that *DOF5.2* acts as a negative regulator of SE. This conclusion is consistent with the observation, that *DOF5.2* expression declines during early somatic embryo formation, compared to explants (0 d). In contrast, overexpression of *bHLH109* resulted in significantly increased SE productivity, in accordance with the fact that *bHLH109* transcript abundance strongly increases during SE (**Figure S2**).

### *AUX/IAA* Genes

**Figure 11:**
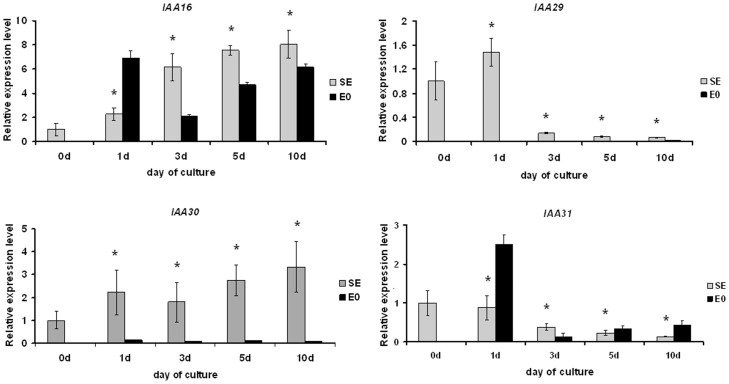
Expression profiles of *AUX/IAA* genes. Shown are expression levels of *AUX/IAA* genes (*IAA16, IAA29, IAA30* and *IAA31*) in explants induced towards alternative morphogenic pathways, i.e. somatic embryogenesis (SE) and seedling development (E0). Values significantly different from E0 are labeled by asterisks (n  = 3; means ± SD are given; Mann-Whitney’s U test; p<0.05).

The *AUX/IAA* genes negatively affecting SE induction potential when mutated (i.e., *IAA16, IAA29, IAA30* and *IAA31*) were subjected to a closer analysis and their transcript levels were evaluated at different time points in cultures derived from the IZE explants. To reveal relations between gene expression and auxin treatment, explants treated with auxin and undergoing SE induction were compared to those of developing seedlings on auxin-free medium. The qRT-PCR analysis indicated that expression patterns during SE varied between the genes; two of the genes (*IAA16* and *IAA30*) were up-regulated while two others (*IAA29* and *IAA31*) were down-regulated during SE (**Figure 11**). Among the *AUX/IAA* genes analysed, *IAA16* displayed the highest increase in transcript level in embryogenic culture. We found that transcript levels of the studied *IAA* genes were significantly influenced by auxin and expression of most of them (*IAA16, IAA29* and *IAA30*) was distinctly stimulated on auxin medium.

## Discussion

### An Extensive Up-regulation of the TF Transcriptome Accompanies SE Induction

This study provides the first, to our knowledge, comprehensive analysis focused on TFs and their expression during the time course of SE. Our analysis indicates that in embryogenically induced somatic tissue of Arabidopsis a large part of the TF transcriptome (over 1,600 TFs) is active. Similarly, over 1,300 TFs were expressed throughout seed development in Arabidopsis and TF genes were found to constitute a much higher fraction (17%) in seed-specific than global (6%) transcriptomes [38]. Thus, tissues undergoing embryogenesis, both in *in planta* and *in vitro*, appear to be highly enriched for TF transcripts supporting the model that regulatory genes have a strong impact on plant developmental processes and in particular, embryogenesis. In support of this, the transcriptome of embryogenesis-related tissues in *Medicago truncatula* includes a high number of TF mRNAs, and 91% vs. 77% of the TF genes were found to be expressed in pods containing developing seeds vs. leaves [32]. Similarly, transcriptome data for reproductive cells in *Brassica napus* showed a distinctly increased number of TF genes expressed in microspores of high embryogenic potency than in non-embryogenic pollen [39].

To identify SE-related TF genes we focused on transcripts differentially expressed during the time course of the embryogenic culture and found that 729 TFs display differential expression in embryogenic culture. Likewise, in shoot organogenesis induced in poplar, 588 TFs (23% of the total) were found differentially expressed [40]. These data reflect the massive genetic reprogramming of somatic cells associated with the induction of new morphogenic paths under *in vitro* conditions and indicate that the control of gene expression at the transcriptional level greatly contributes to the morphogenic switches induced *in vitro*.

Strikingly, when global mRNAs were analysed in embryogenic cultures of other plants much fewer transcripts than found in the present study were reported to be differentially expressed. In rice cultures induced towards different regeneration processes including SE, only 1–3% of the genome was reported to be differentially expressed [41]. Likewise, in soybean and potato 2.6% and 4% of all transcripts were found to be modulated, respectively [25], [26]. The results obtained by global transcriptome analyses suggested a relatively low frequency of differentially expressed TF transcripts [26], [42], [43].

The relatively high number of modulated genes observed in the present study may in part be due to the higher sensitivity of qRT-PCR over hybridization-based approaches, as reported earlier [28], [44]. In accordance with this we identified over twice as many TF mRNAs (1730) in IZE explant tissue than previously discovered (847) by microarrays in the mature green stage of zygotic embryos [38]. Our study furthermore revealed that up-regulation of TF gene expression dominated over down-regulation; up-regulated TFs were almost four times more frequent than down-regulated ones. In ZE, only a moderate predominance (slightly over 50%) of up- over down-regulated mRNAs was observed in early stages of seed development spanning from globular to bent cotyledon embryos [45]. Likewise, recent analysis on several marker genes in pine, including TF mRNAs, documented generally higher gene expression level during SE than during ZE [46].

Similar to our results on the TF transcriptome, global transcriptome analysis in an embryogenic culture of *M. truncatula* indicated a distinct prevalence of up- over down-regulated transcripts [47]. Similarly, differentially expressed genes in cotton embryogenic cultures were also found to be upregulated in most cases [42]. In differentiating embryogenic rice callus, activation of gene expression was more common than repression, but a distinct prevalence of up- versus down-regulated genes was not observed [43]. Few reports indicated that TFs were mostly down-regulated, in contrast to global mRNA profiles [25], [26]. However, the overall relatively small number of TF transcripts detected in these experiments (possibly due to technical limitations associated with microarrays used in those studies) may explain these earlier results.

### TFs Strongly Modulated during SE-induction

The next striking feature of the TF transcriptome during SE induction revealed here was the drastic change (by at least 10-fold) of the expression of almost half (49%) of the modulated transcripts. In contrast, highly up-regulated transcripts were much less frequent in the global ZE transcriptome and constituted only 1–5% of the differentially expressed mRNAs [38]. It can perhaps be assumed that a rapid, massive and strong stimulation of TF expression occurring *in vitro* in SE-induced tissue results from a genome response to auxin treatment. Likewise, in potato, the most dramatic modulation of the transcriptome was observed during the SE induction phase enforced on auxin-containing medium [26], while a drastic fall in gene expression levels was observed in oil palm embryogenic culture after auxin removal from the medium [48].

### Early versus Advanced Stages of SE

Our analysis demonstrated that different TF expression patterns discriminated early from advanced stages of embryogenic culture. In contrast to the embryo induction stage, stabilization of the transcriptome was observed at the more advanced culture stage associated with embryo formation, and most genes (58%) that changed expression by more than 2-fold during the embryo induction stage (i.e., between 0 d and 5 d) retained their expression level thereafter, thus changed expression by less than 2-fold between 5 d and 10 d. Divergent expression profiles were also reported for early and late stages of embryogenesis during seed development [39], [49]. However, data on gene expression profiles specific to different stages of embryogenic cultures are generally scarce. In potato, similar to our results, the differentially expressed transcription-related genes are distinctly less abundant during advanced embryo formation than in the embryo-induction phase [26]. Also studies in maize and *Medicago truncatula* revealed a lower frequency of highly expressed genes in more advanced embryogenic cultures [23], [50].

Apart from distinctly different expression profiles of early and advanced embryogenic cultures, it must be stressed that the great majority (>1,600) of the TFs were expressed across both stages of SE, and the number of TFs exclusively expressed at either the early or advanced SE stage was found to be very small (below 10). Also in ZE, many genes, including TFs, were expressed across multiple embryogenic stages [38], [45] and only a small number of genes was specifically active in each given ZE stage [51], [52]. Likewise, in *Brassica napus*, 30% of the genes expressed in microspore cultures upon embryogenic transition were also associated with developing androgenic embryos [53]. These observations thus indicate an extensive overlap in the transcription regulatory machinery of SE-competent (explant) and SE-responding tissue and that many regulatory genes and their associated biological processes are shared across different stages of embryogenic culture.

### SE-associated TFs

A common approach in screens for SE-associated genes is to contrast transcriptome profiles of embryogenic and non-embryogenic tissues and select the genes differing in expression profiles [22], [25], [50], [54]. This strategy eliminates the genes expressed in response to auxin but not directly involved in the embryogenic switch. A similar approach used here identified 141 genes of distinctly different expression profiles in cultures derived from the highly embryogenic Col-0 accession versus the non-embryogenic *tanmei* mutant. A subset of the 141 genes includes regulators previously found to affect embryogenic development, including sixteen genes reported to be expressed during ZE [38], [45], [49].

Considering the suggested similarities between the genetic programmes governing zygotic and somatic embryogenesis [2], the number of genes required for somatic embryo development was assumed to be convergent to that in ZE. In ZE, the number of genes essential for embryo development in Arabidopsis was estimated to be 500–1000, including 220 *EMB* genes identified as required for normal zygotic embryo development [55], [56], [57]. However, in a recent analysis of the ZE global transcriptome less than 2% of the genes were found to be seed-specific and among them 48 TF genes were reported to be active exclusively, or at elevated levels, in seeds [38]. Strikingly, the majority of the seed-specific TFs [38] were not identified here among the TFs of SE-modulated expression in embryogenic Arabidopsis cultures. We found that only three of them (*ARR19, MYB107, IAA31*) displayed SE-specific expression, whilst 12 other seed-specific TFs were modulated in Col-0 embryogenic culture. This apparently lower than expected similarity between SE- and seed-specific gene expression was also stressed in a study on cucumber embryogenic cultures [58]. In addition, comparative expression profiling of some genes during ZE and SE in pine indicated some differences in the level and pattern of expression, including TF genes [46]. The differences in the gene expression patterns in ZE and SE likely reflect specificities of molecular mechanisms underlying embryogenic development in zygotic vs. somatic cells. Furthermore, the heterogeneity of the cell population analysed in embryogenic cultures may, in contrast to the more homogenous cell populations in ZE, substantially affect the gene expression profiles in tissues undergoing SE.

### Stress-responsive TFs

The induction of SE was considered as a tissue response to stress imposed by *in vitro* culture [59], [60], [61]. In support of this, the activity of many stress-related genes was found to be associated with embryogenic cultures in different plants [25], [47], [48], [50], [62], [63]. Similarly, in our study numerous stress-responsive TFs were expressed in Arabidopsis embryogenic cultures, representing half of the transcripts with SE-specific expression. The great majority (80%) of the stress-related TFs were up-regulated especially at the early stage of SE. Activation of such a large number of stress-related genes during *in vitro* embryo induction is unlikely to indicate a specific mechanism relevant to SE, but rather reflects a general response of the plant´s genome to the environment imposed *in vitro*. A significant proportion (39%) of the stress-related TFs modulated in embryogenic culture belong to the AP2/EREBP, WRKY and NAC families that are commonly activated in response to biotic and abiotic stresses [64], [65], [66], [67].

A massive involvement of TF genes in stress responses can be expected as transcriptional control provides a crucial mechanism of plant responses to various stresses [68]. Several exogenous factors can trigger the expression of stress-related genes under *in vitro* conditions, and 2,4-D used in SE-induction medium is supposed to act as a powerful ‘stressor’ [59], [60], [69]. The strong response of stress-related genes in somatic cells under 2,4-D treatment observed here is in accordance with reports on other plant cultures [25], [26], [70], [71], [72]. Other tissue culture-related conditions can also be expected to influence gene expression *in vitro.* Recently, *WIND1* (*WOUND INDUCED DEDIFFERENTIATION1*) encoding a TF involved in establishment and maintenance of the dedifferentiated status of somatic cells in the absence of exogenous hormones was reported to be activated by tissue wounding [73]. Increased expression of *WIND1* in embryogenic cultures was detected here and in other plant cultures [50], [74].

### Hormone-related TFs

Our analysis revealed a large number of hormone-related TFs that changed their expression during SE, indicating an extensive involvement of hormone-related signaling pathways in this process.

#### Auxin-responsive genes

Auxin is a key trigger of SE in most plants, including Arabidopsis [75]. In accordance with this we observed a large number of auxin-responsive genes to be modulated in Arabidopsis embryogenic culture and similar observations were documented during SE in other plants [26], [41], [43], [76], [77]. Members of the ARF and AUX/IAA transcription regulator/signalling families act in concert to modulate expression of auxin-responsive genes [78], [79]. We found that expression of over half (27/42) of all *AUX/IAA* and *ARF* genes changed during SE in Arabidopsis. In ZE of Arabidopsis, the majority of *AUX/IAA* and *ARF* genes were found active [80], [81]. Transcripts of these genes constituted up to 4% of the seed-specific transcriptome [38] and, as indicated in the present study, *AUX/IAA* and *ARF* transcripts constituted a similar fraction of the SE-associated transcriptome.

Within the group of ARF regulators, *ARF5* (*AT1G19850*) encoding the MONOPTEROS (MP) auxin response factor, was up-regulated in embryogenic cultures of Arabidopsis (this study) and similarly in soybean [25]. *MP* constitutes a key gene in the control of zygotic embryo patterning via affecting polar auxin transport through activation of the auxin efflux carrier gene *PIN1* [82]. Significant activity of *MP* in embryogenic cultures may indicate that, similar to ZE, polar auxin transport and patterning are associated with somatic embryo induction and development. In support of this, mutations in both, *MP* and *TIR1* (*TRANSPORT INHIBITOR RESPONSE1*) were found to partly impair SE induction in Arabidopsis IZE explants (Malgorzata D. Gaj and A. Trojanowska, unpublished data). An important role of polar auxin transport for proper embryogenesis is supported by the fact that embryo development is impaired *in vivo* [83] and *in vitro* [84], [85], [86], [87] when auxin transport is disturbed.

We also observed an upregulation of several other *ARF* genes in embryogenic cultures, including *ARF6*, *ARF8*, *ARF16* and *ARF17*. We found *ARF6* to be co-expressed with *ARF8*, similarly to what has been reported for ZE [45], [49]. *ARF8* has been suggested to control the level of free IAA (indole-3-acetic acid) in a negative feedback fashion by regulating expression of *GH3* genes [88]. Expression of *ARF16* and *ARF17* was also modulated during ZE [45], [49]. *ARF17* has been implicated as a regulator of GH3-like early auxin response genes [89]. *ARF16* together with *ARF10* and *IAA17/AXR3* regulate distal stem cell differentiation in Arabidopsis roots acting upstream of *PLETHORA* (*PLT*) [90]. Of note, these genes (*ARF10, ARF16, IAA17, PLT1* and *PLT2*) were up-regulated in embryogenic Arabidopsis cultures.

Similar to *ARFs,* reports on *AUX/IAA* expression in embryogenic cultures of plants are rare; of note, however, homologs of the Arabidopsis *IAA9* and *IAA8* genes were found expressed during SE in *Cyclamen persicum* and *Gossypium hirsutum* [91], [92]. In the present analysis almost 70% of the *AUX/IAA* family members displayed modulated expression in embryogenic cultures suggesting their involvement in SE. In support of this we found *iaa* mutants (*iaa16, iaa29, iaa30* and *iaa31*) to be significantly impaired in the embryogenic response.

#### *AP2/EREBP* TFs and ethylene responses

The SE-modulated TF transcriptome was highly enriched for members of the AP2/EREBP family. Numerous *AP2/EREBP* genes were previously shown to control SE and shoot organogenesis *in vitro*, and several members of the family were reported to promote embryo development in somatic tissues when overexpressed, including e.g. *BABY BOOM* (*BBM*) [13], *AGAMOUS-LIKE15* (*AGL15*) [15], [93] and *EMBRYOMAKER* (*EMK*) [19]. Expression of *AP2/EREBP* TFs was frequently found to be modulated in embryogenic cultures of different plants [25], [50], [76], [77], [94], [95] including Arabidopsis (this report). Many members of the ERF subfamily are involved in ethylene responses [65]. Hence, enhanced expression of *AP2/EREBP* genes during the *in vitro* culture may reflect a general stress response of the tissues as e.g. induced by wounding or hormonal treatment [68], while some *ERF* genes may be specifically involved in the induction of SE. The role of ethylene for somatic embryo development was demonstrated in *Medicago truncatula*, where *SOMATIC EMBRYO-RELATED FACTOR1* (*MtSERF1*), an ERF subfamily TF affecting ethylene biosynthesis, is crucial for embryo induction [50]. Likewise, in *Pinus silvestris* an increased content of endogenous ethylene appears to be required for somatic embryo development [95]. Recently, ethylene biosynthesis and perception were also reported to be involved in SE induction in Arabidopsis [96]. In support of this, the extensive modulation of many (49) ethylene-related TFs of the ERF, MYB, bHLH, NAC and WRKY families was observed here for embryogenic Col-0 cultures, and mutations affecting *ERF022* (encoding an ERF TF**;** **Figure 10**) and *ACC SYNTHASE4* (*ACS4*; involved in ethylene biosynthesis) appeared to significantly decrease explant capacity for SE (data not shown). Our preliminary analysis indicates regulatory relationships between ERF022 and genes acting in ethylene signaling and biosynthesis (Katarzyna Nowak and Malgorzata D. Gaj, unpublished). Another ethylene related gene, *RAP2.6L* (*RELATED TO AP2 6L*; *AT5G13330*) of the AP2/EREBP family, was found here to be up-regulated in embryogenic cultures. *RAP2.6L* expression is also induced during shoot organogenesis [94], in proliferating cells of newly formed tissues after wounding, and by stress hormones and abiotic stresses [97], [98].

#### Cytokinin-related TFs

Although cytokinin is not included in SE-induction medium, the involvement of cytokinin-related TFs in embryogenic development may be expected due to widespread crosstalk between auxin and cytokinin signalling [99], [100], [101], [102]. We here observed 16 cytokinin response-associated TFs to be affected in the auxin-induced embryogenic Col-0 cultures, including key cytokinin regulatory genes, i.e. *CYTOKININ RESPONSE FACTORS* (*CRFs*) and *Arabidopsis RESPONSE REGULATORS* (*ARRs*). Of eight *CRFs*, four (*CRF2, 3, 4* and *5*) were up-regulated in Col-0 embryogenic cultures. CRFs mediate a large fraction of the transcriptional response to cytokinin to regulate development of embryos, cotyledons, and leaves and they function together with type-B ARRs [103]. Two type-B *ARR* genes, i.e. *ARR19* and *ARR10*, had altered expression in Col-0 cultures. *ARR10* transcripts were up-regulated in early and advanced stages of SE and similarly, up-regulation of the *ARR10* homolog *MtRR1* (*Mtr.43735.1*) was reported in embryogenic cultures of *M. truncatula* [47]. *ARR10*, together with *ARR1* and *ARR12*, is proposed to play a general role in cytokinin signal transduction [104].

#### Gibberellin-related TFs

In Arabidopsis, the endogenous level of gibberellins in somatic tissue seems to be negatively correlated with embryogenic potential. The *lec* mutants, displaying increased GA content [105], were found to have a drastically reduced ability for SE [106]. Similarly, the *pickle* mutant which has elevated levels of bioactive GAs displays reduced embryogenic potential in cultures of IZEs, and exogenously supplied GA_3_ was demonstrated to decrease tissue capacity for SE induction [106].

In support of the inhibitory effect of GA on embryogenic capacity in Arabidopsis, several genes important for the negative regulation of GA responses were found to display an SE-specific up-regulation, including the DELLA-encoding genes *RGL1* (*RGA-LIKE1,* RGA for repressor of ga1-3) and *RGL2*. DELLA proteins interact with multiple environmental and hormonal response pathways and restrain plant growth [107]. The stimulation of DELLA-encoding genes in Col-0 embryogenic cultures may also be associated with stress responses as DELLA accumulation was reported to elevate the expression of genes encoding ROS detoxification enzymes, thus reducing ROS levels [108]. Another suppressor of GA responses, *SHORT INTERNODES* (*SHI*), was found to be up-regulated in Col-0 embryogenic cultures; in intact plants, *SHI* affects the development of shoot and root primordia [109].

### Role of TFs in SE

To increase the probability of finding TFs functionally relevant for SE, we included the *tanmei* mutant in our transcriptome analysis. As *tanmei* lacks the capacity for SE, TFs differentially expressed between Col-0 and the mutant may represent candidate regulators of SE, although genes not specifically associated with SE may also be expressed at different levels in the two genetic backgrounds. Considering the results of our global expression analysis we selected 21 genes (18 of which showed altered expression in Col-0 *vs*. *tanmei*, and three genes displayed differential expression in embryogenic culture) to test their potential relevance for somatic embryo formation, using T-DNA insertion mutants and transgenic lines expression the TFs under the control of a ß-estradiol-inducible promoter [110]. The majority (70%) of the mutants analyzed were significantly impaired in their SE capacity suggesting an involvement of the tested TFs in this process.

We found that various T-DNA insertion lines impaired in SE were actually mutated in genes related to stress responses, including *ERF022, NTL8, DREB2F, ATHB*-*12*, *LBD20* and *MYB74*. Mutating *ERF022* increases the plant´s sensitivity to osmotic and salinity stress, whilst overexpressing it triggers the opposite phenotype (Katarzyna Nowak and Malgorzata D. Gaj, data not shown). *NTL8* of the NAC TF family was reported to regulate gibberellic acid-mediated salt signalling during Arabidopsis seed germination [111]. Expression of *DREB2F* is affected by abiotic and biotic stresses (eFP browser: http://www.bar.utoronto.ca/efp/cgi). *ATHB12* together with *ATHB7* was reported to encode a potential regulator of growth in response to water deficit [112]. *LBD20 (LOB DOMAIN-CONTAINING PROTEIN20*) has recently been suggested to be involved in transcriptional regulation of plant defence responses against pest or pathogen attack [113]. *MYB74* is a close homolog of *MYB102* which was demonstrated to be induced by osmotic stress and wounding [114]. Summarizing, the SE-impaired phenotypes observed in mutants of stress-related genes strongly support the notion that SE induction shares, at the molecular level, processes that are also relevant to general stress responses.

In contrast to the insertion mutants, phenotypes of transgenic lines overexpressing TFs under the control of a chemically inducible promoter were generally less informative. However, for two TFs, i.e. *DOF5.2* and *bHLH109*, we observed a clear function in SE. The phenotype observed upon induced overexpression of *DOF5.2* (reduced SE capacity) together with the fact that expression of the gene decreases during early stages of somatic embryogenesis suggests that DOF5.2 functions as a negative regulator of SE induction. Currently, the exact molecular function of *DOF5.2* is unknown, however, the gene was shown to be specifically expressed in the quiescent centre of roots and a role for stem cell niche maintenance in the root meristem possibly by affecting auxin flux was postulated [115].

The other gene found to affect SE is *bHLH109,* which in contrast to *DOF5.2* appears to act as a positive regulator of somatic embryo formation. Accordingly, expression of *bHLH109* was found to be highly upregulated in embryogenic cultures, and auxin strongly enhanced its expression. Identifying the downstream target genes controlled by bHLH109 will help to better understand through which regulatory networks the bZIP TF promotes embryogenic development in the future.

## Conclusions

Our study provides the first comprehensive analysis of the global TF transcriptome of plant somatic tissue undergoing embryogenic induction during *in vitro* culture. TF genes of drastically different expression in embryogenic vs. non-embryogenic cultures were selected as candidates for further studies aiming at the characterization of genes with decisive roles in SE.

The results presented here indicate the presence of a regulatory burst at the gene expression level that is associated with early stages of somatic embryo development. The global TF transcriptome associated with SE induction reflects the combinational effects of stress and hormone signalling related to the *in vitro* environment imposed during culture. Accordingly, among the TFs showing SE-specific expression those involved in stress and hormone responses, plant and especially flower development were found most frequent. The use of Arabidopsis for this study opens new avenues for advanced analysis of the selected SE- associated candidate genes based on genomic data, mutant collections, transgenic lines and other genomic tools available for this model species. The study provides guidelines for further research on functional genomics of SE.

## Materials and Methods

### Plant Material and Growth Conditions

Two *Arabidopsis thaliana* (L.) Heynh. genotypes of different embryogenic capacity were analyzed, i.e. the highly embryogenic Col-0 ecotype and the SE-impaired *tanmei* (*tan1-2)* mutant [34]. Additionally, mutants carrying T-DNA insertions [64] in selected TF genes were analysed with respect to their capacity for somatic embryo formation. The parental Col-0 ecotype and the insertion mutants were obtained from NASC (The Nottingham Arabidopsis Stock Center; http://arabidopsis.info/). T-DNA insertion lines (**Table S4**) originated from the SALK and SAIL collections; homozygous plants carrying insertions in TF genes were selected from a segregating T_3_ population according to standard procedures. Seeds of the *tan1-2* mutant were kindly provided by J. J. Harada (University of California, Davis, USA). Plants were grown in Jiffy-7 peat pots of 42 mm diameter (Jiffy) in a ‘walk-in’ type phytotron, under controlled conditions: 22°C, 16h/8h (light/dark), 100 µE/m^2^s light intensity.

### Estradiol-inducible TF Overexpression Lines

To generate transgenic plants expressing TFs under the control of an estradiol-inducible promoter, the coding regions of the selected genes (*NTL8, ERF022, bHLH89, bHLH109, REM22, AGL2, WRKY31, DOF5.2*) were amplified by PCR from Arabidopsis leaf or zygotic embryo cDNA using primers IOE-fwd and IOE-rev (**Table S5**), inserted into pBluescript SK (Stratagene) and then cloned via *Xho*I (or *Asc*I) and *Spe*I sites into the pER8 vector [110]. *Agrobacterium tumefaciens* strain GV3101 was used for *A. thaliana* (Col-0) transformation. Seedlings of selected homozygous transgenic lines were used for expression analysis. RNA was isolated (TriPure Reagent; Roche) from ß-estradiol-treated (5 µM, 2 d) and mock-treated (0.01% ethanol) seedlings, and cDNA was synthesized using RevertAid First Strand cDNA Synthesis Kit (Fermentas). The resulting cDNA was used for qRT-PCR (**Table S6**). LightCycler Fast-Start DNA Master SYBR Green I (Roche) and appropriate primers were used for qRT-PCR reactions.

### Induction of Somatic Embryogenesis

A standard protocol was used to induce somatic embryogenesis in Arabidopsis under *in vitro* conditions [116]. In brief, explants, i.e., immature zygotic embryos (IZEs) at the late cotyledonary stage of development, were excised from siliques 10–12 days after pollination. Siliques were surface-sterilized with sodium hypochlorite (20% commercial bleach) and washed thoroughly with sterile water. Then IZEs were isolated and placed on E5 solid medium containing B5 salts and vitamins [117] and supplemented with 5 µM 2,4-D, 20 g l^-1^ sucrose and 3.5 g l^-1^ Phytagel (Sigma). To induce overexpression of TFs in pER8-TF-transformed transgenic cultures, E5 medium was supplemented with 5 µM of ß-estradiol.

Cultures were maintained in the controlled conditions of a growth chamber: 22°C, 16h/8h (light/dark), light intensity 50 µE/m^2^ s. At selected time points of the culture (0, 5 and 10 d), explants of Col-0 and *tan1-2* were sampled for transcriptome analysis.

The capacity for SE in T-DNA insertion mutants and transgenic lines overexpressing TFs was evaluated after 21 days of *in vitro* culture. Embryogenic potential of mutants and transgenic lines was evaluated by calculation of SE efficiency (i.e., the percentage of explants forming somatic embryos) and SE productivity (i.e., the average number of somatic embryos produced per SE-responding explant). SE efficiency and productivity of the analysed genotypes was compared to Col-0-derived cultures. All experiments were conducted in three independent replicates, and at least 30 explants (10 explants/Petri dish) were analysed per replicate.

### Statistical Analysis

Kruskal-Wallis ANOVA rank and Mann-Whitney’s U statistical tests were applied to calculate significant differences (at p  = 0.05) between combinations.

### Transcriptome Profiling by Multi-parallel qRT-PCR

Quantitative RT-PCR was used to compare the expression levels of 1,880 Arabidopsis TF genes in the SE cultures of Col-0 and *tan1–2*. Total RNA was isolated at 0, 5 and 10 d of wild-type- (WT) and mutant-derived cultures, using RNAqueous kit (Ambion). The isolates were digested with Turbo DNA-free kit (Ambion) to remove DNA contaminants. SuperScript III reverse transcriptase (Invitrogen) was used for cDNA synthesis. qRT-PCR was done as described [31], [118], [119]. PCR reactions were run on an ABI PRISM 7900 HT sequence detection system (Applied Biosystems Applera, Darmstadt, Germany).

Data analysis was performed using SDS 2.2.1 software (Applied Biosystems). All amplification curves were analysed with a normalized reporter (R_n_: the ratio of the fluorescence emission intensity of SYBR Green to the fluorescence signal of the passive reference dye) threshold of 0.3 to obtain the C_T_ (threshold cycle) values. Four replicates of the reference control gene, *UBQ* (*AT1G55060*), were measured in each PCR run, and their median C_T_ was used for relative expression analyses. Expression data were submitted to the NCBI Gene Expression Omnibus (GEO) repository (www.ncbi.nlm.nih.gov/geo/) under accession number GSE45697.

To find significant changes between the genotypes (Col-0 and *tan1–2*) and the time points, ANOVA followed by false discovery rate (FDR) correction was applied using a custom R script (http://www.r-project.org). Only TFs which displayed an FDR corrected p-value<0.05 were considered for further analysis. Furthermore, different comparisons between genotypes and time points were performed using Studentś *t*-test (p<0.05). The analysis was performed in two ways: (1) to identify differentially expressed TFs that are specific for the different time points in Col-0, and (2) to identify TFs differentially expressed between Col-0 and *tan1–2* at each time point. The fold change was calculated using (2)^–ΔΔC^_T_, where ΔΔC_T_ represents ΔC_T reference condition_ − ΔC_T compared condition_. The obtained results were transformed to log_2_ scale. Candidates were extracted using thresholds of 2- and 10-fold change.

Principal component analysis (PCA) was performed using the prcomp function of the “stats” package in R [120].

## Supporting Information

Figure S1 **TF families among differentially expressed and SE-associated genes.** For each TF family the percentage of genes differentially expressed or being SE-associated is indicated.
(TIF)

Figure S2 **Expression levels of** ***bHLH109*** **and** ***DOF5.2*** **TFs in explants induced towards alternative morphogenic pathways, i.e. somatic embryogenesis (SE) and seedling development (E0).** Values significantly different from E0 are marked by asterisks (n  = 3; means ± SD are given; Mann-Whitney’s U test; p<0.05).
(TIF)

Table S1 **TFs exclusively or highly expressed in embryogenic Col-0 explants compared to non-embryogenic** ***tanmei*** **mutant explants.**
(DOC)

Table S2 **Expression values of 729 TFs modulated in Col-0 embryogenic culture.**
(XLS)

Table S3 **TFs showing an at least 10-fold expression change during early culture stages.**
(DOC)

Table S4 **T-DNA insertion lines used for the functional analysis of selected TFs.**
(DOC)

Table S5 **Primers used for the amplification of open reading frames.**
(DOC)

Table S6 **Expression level of transgenes in seedlings treated with ß-estradiol (5 µM) for 2 days.**
(DOC)

## References

[1] Costa S, Shaw P (2007) „Open minded” cells: how cells can change fate. Trends Cell Biol 17: 101–106. 17194589 10.1016/j.tcb.2006.12.005

[2] Zimmerman JL (1993) Somatic embryogenesis: a model for early development in higher plants. Plant Cell 5: 1411–1423. 12271037 10.1105/tpc.5.10.1411 PMC160372

[3] Dodeman VL, Ducreux G, Kreis M (1997) Zygotic embryogenesis versus somatic embryogenesis. J Exp Bot 48: 1493–1509.

[4] Long TA, Benfey PN (2006) Transcription factors and hormones: new insights into plant cell differentiation. Curr Opin Cell Biol 18: 710–714. 17034999 10.1016/j.ceb.2006.09.004

[5] Riechmann JL, Ratcliffe OJ (2000) A genomic perspective on plant transcription factors. Curr Opin Plant Biol 3: 423–434. 11019812 10.1016/s1369-5266(00)00107-2

[6] Zhang JZ (2003) Overexpression analysis of plant transcription factors. Curr Opin Plant Biol 6: 1–11. 10.1016/s1369-5266(03)00081-5 12972043

[7] Zeitlinger J, Stark A (2010) Developmental gene regulation in the era of genomics. Dev Biol 339: 230–239. 20045679 10.1016/j.ydbio.2009.12.039

[8] Aoi T, Yae K, Nakagawa M, Ichisaka T, Okita K, et al (2008) Generation of pluripotent stem cells from adult mouse liver and stomach cells. Sci 321(5889): 699–702. 10.1126/science.1154884 18276851

[9] Jaenisch R, Young R (2008) Stem cells, the molecular circuitry of pluripotency and nuclear reprogramming. Cell 132: 567–582. 18295576 10.1016/j.cell.2008.01.015 PMC4142810

[10] Patel M, Yang S (2010) Advances in reprogramming somatic cells to induced pluripotent stem cells. Stem Cell Rev 6: 367–380. 20336395 10.1007/s12015-010-9123-8 PMC2924949

[11] Takahashi K, Tanabe K, Ohnuki M, Narita M, Ichisaka T, et al (2007) Induction of pluripotent stem cells from adult human fibroblasts by defined factors. Cell 131: 861–872. 18035408 10.1016/j.cell.2007.11.019

[12] Mitsuda N, Ohme-Takagi M (2009) Functional analysis of transcription factors in Arabidopsis. Plant Cell Physiol 50: 1232–1248. 19478073 10.1093/pcp/pcp075 PMC2709548

[13] Boutilier K, Offringa R, Sharma VK, Kieft H, Ouellet T, et al (2002) Ectopic expression of *BABY BOOM* triggers a conversion from vegetative to embryonic growth. Plant Cell 14: 1737–1749. 12172019 10.1105/tpc.001941 PMC151462

[14] Zuo J, Niu QW, Frugis G, Chua NH (2002) The *WUSCHEL* gene promotes vegetative-to-embryonic transition in Arabidopsis. Plant J 30: 349–359. 12000682 10.1046/j.1365-313x.2002.01289.x

[15] Harding EW, Tang W, Nichols KW, Fernandez DE, Perry SP (2003) Expression and maintenance of embryogenic potential is enhanced through constitutive expression of *AGAMOUS-Like 15* . Plant Physiol 133: 653–663. 14512519 10.1104/pp.103.023499 PMC219041

[16] Gaj MD, Zhang S, Harada JJ (2005) Leafy cotyledon genes are essential for induction of somatic embryogenesis of Arabidopsis. Planta 222: 977–988. 16034595 10.1007/s00425-005-0041-y

[17] Yamamoto A, Kagaya Y, Toyoshima R, Kagaya M, Takeda S, et al (2009) Arabidopsis NF-YB subunits LEC1 and LEC1-LIKE activate transcription by interacting with seed-specific ABRE-binding factors. Plant J 58: 843–856. 19207209 10.1111/j.1365-313X.2009.03817.x

[18] Wang X, Niu QW, Teng C, Li C, Mu J, et al (2009) Overexpression of *PGA37/MYB118* and *MYB115* promotes vegetative-to-embryonic transition in Arabidopsis. Cell Res 19: 224–235. 18695688 10.1038/cr.2008.276

[19] Tsuwamoto R, Yokoi S, Takahata Y (2010) Arabidopsis *EMBRYOMAKER* encoding an AP2 domain transcription factor plays a key role in developmental change from vegetative to embryonic phase. Plant Mol Biol 73: 481–492. 20405311 10.1007/s11103-010-9634-3

[20] Stone SL, Kwong LW, Yee KM, Pelletier J, Lepiniec L, et al (2001) *LEAFY COTYLEDON2* encodes a B3 domain transcription factor that induces embryo development. Proc Natl Acad Sci USA 98: 11806–11811. 11573014 10.1073/pnas.201413498 PMC58812

[21] van Zyl L, Bozhkov PV, Chacham D, Sederoff RR, von Arnold S (2003) Up, down and up again is a signature global gene expression pattern at the beginning of gymnosperm embryogenesis. Gene Expr Patterns 3: 83–91. 12609608 10.1016/s1567-133x(02)00068-6

[22] Stasolla C, Belmonte MF, van Zyl L, Craig DL, Liu W, et al (2004) The effect of reduced glutathione on morphology and gene expression of white spruce (*Picea glauca*) somatic embryos. J Exp Bot 55: 695–709. 14966213 10.1093/jxb/erh074

[23] Che P, Love TM, Frame BR, Wang K, Carriquiry AL, et al (2006) Gene expression patterns during somatic embryo development and germination in maize Hi II callus cultures. Plant Mol Biol 62: 1–14. 16845483 10.1007/s11103-006-9013-2

[24] Taguchi-Shiobara F, Yamamoto T, Yano M, Oka S (2006) Mapping QTLs that control the performance of rice tissue culture and evaluation of derived near-isogenic lines. Theor Appl Genet 112: 968–976. 16418858 10.1007/s00122-005-0200-3

[25] Thibaud-Nissen F, Shealy RT, Khanna A, Vodkin LO (2003) Clustering of microarray data reveals transcript patterns associated with somatic embryogenesis in soybean. Plant Physiol 132: 118–136. 12746518 10.1104/pp.103.019968 PMC166958

[26] Sharma KS, Millam S, Hedley PE, McNicol S, Bryan GJ (2008) Molecular regulation of somatic embryogenesis in potato: an auxin led perspective. Plant Mol Biol 68: 185–201. 18553172 10.1007/s11103-008-9360-2

[27] Horak CE, Snyder M (2002) Global analysis of gene expression in yeast. Funct Integr Genomics 2: 171–180. 12192590 10.1007/s10142-002-0065-3

[28] Czechowski T, Bari RP, Stitt M, Scheible WR, Udvardi MK (2004) Real Time RT-PCR profiling of over 1400 Arabidopsis transcription factors: unprecedented sensitivity reveals novel root- and shoot-specific genes. Plant J 38: 366–379. 15078338 10.1111/j.1365-313X.2004.02051.x

[29] Osuna D, Usadel B, Morcuende R, Gibon Y, Bläsing OE, et al (2007) Temporal responses of transcripts, enzyme activities and metabolites after adding sucrose to carbon-deprived Arabidopsis seedlings. Plant J 49: 463–491. 17217462 10.1111/j.1365-313X.2006.02979.x

[30] Morcuende R, Bari R, Gibon Y, Zheng W, Pant BD, et al (2007) Genome-wide reprogramming of metabolism and regulatory networks of Arabidopsis in response to phosphorus. Plant Cell Environ 30: 85–112. 17177879 10.1111/j.1365-3040.2006.01608.x

[31] Caldana C, Scheible WR, Mueller-Roeber B, Ruzicic S (2007) A quantitative RT-PCR platform for high-throughput expression profiling of 2500 rice transcription factors. Plant Methods 3: 7. 17559651 10.1186/1746-4811-3-7 PMC1914063

[32] Kakar K, Wandrey M, Czechowski T, Gertner T, Scheible WR, et al (2008) A community resource for high-throughput quantitative RT-PCR analysis of transcription factor gene expression in *Medicago truncatula* . Plant Methods 4: 18. 18611268 10.1186/1746-4811-4-18 PMC2490690

[33] Rohrmann J, Tohge T, Alba R, Osorio S, Caldana C, et al (2011) Combined transcription factor profiling, microarray analysis and metabolite profiling reveals the transcriptional control of metabolic shifts occurring during tomato fruit development. Plant J 68: 999–1013. 21851430 10.1111/j.1365-313X.2011.04750.x

[34] Baster P, Ledwoń A, Gliwicka M, Trojanowska A, Gaj MD (2009) Arabidopsis *tanmei*/*emb2757* embryo mutant is defective for *in vitro* plant morphogenesis. Plant Cell Tiss Org Cult 99: 305–312.

[35] Kurczyńska EU, Gaj MD, Ujczak A, Mazur E (2007) Histological analysis of direct somatic embryogenesis in *Arabidopsis thaliana* (L.) Heynh. Planta 226: 619–626. 17406890 10.1007/s00425-007-0510-6

[36] Yamagishi K, Nagata N, Matsudaira Yee K, Braybrook SA, Pelletier J, et al (2005) *TANMEI/EMB2757* encodes a WD repeat protein required for embryo development in Arabidopsis. Plant Physiol 139: 163–173. 16113228 10.1104/pp.105.060467 PMC1203366

[37] Nezames CD, Sjogren CA, Barajas JF, Larsen PB (2012) The Arabidopsis cell cycle checkpoint regulators TANMEI/ALT2 and ATR mediate the active process of aluminum-dependent root growth inhibition. Plant Cell 24: 608–621. 22345493 10.1105/tpc.112.095596 PMC3315236

[38] Le BH, Cheng C, Bui AQ, Wagmaister JA, Henry KF, et al (2010) Global analysis of gene activity during Arabidopsis seed development and identification of seed-specific transcription factors. Proc Natl Acad Sci USA 7: 8063–8070. 10.1073/pnas.1003530107 PMC2889569 20385809

[39] Whittle CA, Malik MR, Li R, Krochko JE (2010) Comparative transcript analyses of the ovule, microspore, and mature pollen in *Brassica napus* . Plant Mol Biol 72: 279–299. 19949835 10.1007/s11103-009-9567-x

[40] Bao Y, Dharmawardhana P, Mockler DC, Strauss SH (2009) Genome scale transcriptome analysis of shoot organogenesis in *Populus* . BMC Plant Biol 9: 132–147. 19919717 10.1186/1471-2229-9-132 PMC2784466

[41] Su N, He K, Jiao Y, Chen C, Zhou J, et al (2007) Distinct reorganization of the genome transcription associates with organogenesis of somatic embryo, shoots, and roots in rice. Plant Mol Biol 63: 337–349. 17072560 10.1007/s11103-006-9092-0

[42] Zeng F, Zhang X, Zhu L, Tu L, Guo X, et al (2006) Isolation and characterization of genes associated to cotton somatic embryogenesis by suppression subtractive hybridization and macroarray. Plant Mol Biol 60: 167–183. 16429258 10.1007/s11103-005-3381-x

[43] Chakrabarty D, Trivedi KP, Shri M, Misra P, Asif MH, et al (2010) Differential transcriptional expression following thidiazuron-induced callus differentiation developmental shifts in rice. Plant Biol 12: 46–59. 20653887 10.1111/j.1438-8677.2009.00213.x

[44] Busch W, Lohmann JU (2007) Profiling a plant: expression analysis in Arabidopsis. Curr Opin in Plant Biol 10: 136–141. 17291825 10.1016/j.pbi.2007.01.002

[45] Xiang D, Venglat P, Tibiche C, Yang H, Risseeuw E, et al (2011) Genome-wide analysis reveals gene expression and metabolic network dynamics during embryo development in Arabidopsis. Plant Physiol 156: 346–356. 21402797 10.1104/pp.110.171702 PMC3091058

[46] Lara-Chavez A, Egertsdotter U, Flinnnajlepiej BS (2012) Comparison of gene expression markers during zygotic and somatic embryogenesis in pine. In Vitro Cell Dev Biol - Plant 48: 341–354.

[47] Imin N, Goffard N, Nizamidin M, Rolfe BG (2008) Genome-wide transcriptional analysis of super-embryogenic *Medicago truncatula* explant cultures. BMC Plant Biol 8: 110. 18950541 10.1186/1471-2229-8-110 PMC2605756

[48] Lin HC, Morcillo F, Dussert S, Tranchant-Dubreuil C, Tregear JW, et al (2009) Transcriptome analysis during somatic embryogenesis of the tropical monocot *Elaeis guineensis*: evidence for conserved gene functions in early development. Plant Mol Biol 70: 173–192. 19199047 10.1007/s11103-009-9464-3

[49] Spencer MWB, Casson SA, Lindsey K (2007) Transcriptional profiling of the Arabidopsis embryo. Plant Physiol 143: 924–940. 17189330 10.1104/pp.106.087668 PMC1803724

[50] Mantiri FR, Kurdyukov S, Lohar DP, Sharopova N, Saeed NA, et al (2008) The transcription factor MtSERF1 of the ERF subfamily identified by transcriptional profiling is required for somatic embryogenesis induced by auxin plus cytokinin in *Medicago truncatula* . Plant Physiol 146: 1622–1636. 18235037 10.1104/pp.107.110379 PMC2287338

[51] Casson S, Spencer M, Walker K, Lindsey K (2005) Laser capture microdissection for the analysis of gene expression during embryogenesis of Arabidopsis. Plant J 42: 111–123. 15773857 10.1111/j.1365-313X.2005.02355.x

[52] Harada JJ, Pelletier JM (2012) Genome-wide analyses of gene activity during seed development. Seed Sci Res 22: S15–S22.

[53] Malik MR, Wang F, Dirpaul JM, Zhou N, Polowick PL, et al (2007) Transcript profiling and identification of molecular markers for early microspore embryogenesis in *Brassica napus* . Plant Physiol 144: 134–154. 17384168 10.1104/pp.106.092932 PMC1913795

[54] Low ET, Alias H, Boon SH, Shariff EM, Tan CY, et al (2008) Oil palm (*Elaeis guineensis* Jacq.) tissue culture ESTs: Identifying genes associated with callogenesis and embryogenesis. BMC Plant Biol 8: 62. 18507865 10.1186/1471-2229-8-62 PMC2442076

[55] Franzmann LH, Yoon ES, Meinke DW (1995) Saturating the genetic map of *Arabidopsis thaliana* with embryonic mutations. Plant J 7: 341–350.

[56] McElver J, Tzafrir I, Aux G, Rogers R, Ashby C, et al (2001) Insertional mutagenesis of genes required for seed development in *Arabidopsis thaliana* . Genetics 159: 1751–1763. 11779812 10.1093/genetics/159.4.1751 PMC1461914

[57] Tzafrir I, Pena-Muralla R, Dickerman A, Berg M, Rogers R, et al (2004) Identification of genes required for embryo development in Arabidopsis. Plant Physiol 135: 1206–1220. 15266054 10.1104/pp.104.045179 PMC519041

[58] Wis?niewska A, Grabowska A, Pietraszewska-Bogiel A, Tagashira N, Zuzga S, et al (2012) Identification of genes up-regulated during somatic embryogenesis of cucumber. Plant Physiol Biochem 50: 54–64. 22099519 10.1016/j.plaphy.2011.09.017

[59] Fehér A, Pasternak TP, Dudits D (2003) Transition of somatic plant cells to an embryogenic state. Plant Cell Tiss Org Cult 74: 201–228.

[60] Karami O, Saidi A (2010) The molecular basis for stress-induced acquisition of somatic embryogenesis. Mol Biol Rep 37: 2493–2507. 19705297 10.1007/s11033-009-9764-3

[61] Zavattieri MA, Frederico AM, Lima M, Sabino R, Arnholdt-Schmitt B (2010) Induction of somatic embryogenesis as an example of stress-related plant reactions. Electronic J Biotech 13: 1–9.

[62] Domoki M, Györgyey J, Bíró J, Pasternak TP, Zvara A, et al (2006) Identification and characterization of genes associated with the induction of embryogenic competence in leaf-protoplast-derived alfalfa cells. Biochem Biophys Acta 1759: 543–551. 17182124 10.1016/j.bbaexp.2006.11.005

[63] Sun L, Wu Y, Su S, Liu H, Yang G, et al (2012) Differential gene expression during somatic embryogenesis in the maize (*Zea mays* L.) inbred line H99. Plant Cell Tiss Org Cult 109: 271–286.

[64] Alonso JM, Stepanova AN, Leisse TJ, Kim CJ, Chen H, et al (2003) Genome-wide insertional mutagenesis of *Arabidopsis thaliana* . Sci 301: 653–657. 10.1126/science.1086391 12893945

[65] Nakano T, Suzuki K, Fujimura T, Shinshi H (2006) Genome-wide analysis of the ERF gene family in Arabidopsis and rice. Plant Physiol 140: 411–432. 16407444 10.1104/pp.105.073783 PMC1361313

[66] Grafi G, Chalifa-Caspi V, Nagar T, Plaschkes I, Barak S, et al (2011) Plant response to stress meets dedifferentiation. Planta 233: 433–438. 21312042 10.1007/s00425-011-1366-3

[67] Li HW, Zang BS, Deng XW, Wang XP (2011) Overexpression of the trehalose-6-phosphate synthase gene *OsTPS1* enhances abiotic stress tolerance in rice. Planta 234: 1007–1018. 21698458 10.1007/s00425-011-1458-0

[68] Singh KB, Foley RC, Onate-Sanchez L (2002) Transcription factors in plant defense and stress responses. Curr Opin Plant Biol 5: 430–436. 12183182 10.1016/s1369-5266(02)00289-3

[69] Fehér A (2008) The initiation phase of somatic embryogenesis: what we know and what we don’t. Acta Biol Szegediensis 52: 53–56.

[70] Puigderrajols P, Jofre A, Mir G, Pla M, Verdaguer D, et al (2002) Developmentally and stress-induced small heat shock proteins in cork oak somatic embryos. J Exp Bot 53: 1445–1452. 12021292

[71] Nolan KE, Saeed NA, Rose RJ (2006) The stress kinase gene MtSK1 in *Medicago truncatula* with particular reference to somatic embryogenesis. Plant Cell Rep 25: 711–722. 16518633 10.1007/s00299-006-0135-4

[72] Park JE, Park JY, Kim YS, Staswick PE, Jeon J, et al (2007) GH3-mediated auxin homeostasis links growth regulation with stress adaptation response in Arabidopsis. J Biol Chem 282: 10036–10046. 17276977 10.1074/jbc.M610524200

[73] Iwase A, Mitsuda N, Koyama T, Hiratsu K, Kojima M, et al (2011) The AP2/ERF transcription factor WIND1 controls cell dedifferentiation in Arabidopsis. Curr Biol 21: 508–514. 21396822 10.1016/j.cub.2011.02.020

[74] Grabowska A, Wisniewska A, Tagashira N, Malepszy S, Filipecki M (2009) Characterization of *CsSEF1* gene encoding putative CCCH type zinc finger protein expressed during cucumber somatic embryogenesis. J Plant Physiol 166: 310–323. 18778873 10.1016/j.jplph.2008.06.005

[75] Gaj MD (2004) Factors influencing somatic embryogenesis induction and plant regeneration with particular reference to *Arabidopsis thaliana* (L.) Heynh. Plant Growth Regul 43: 27–47.

[76] Legrand S, Hendriks T, Hilbert JL, Quillet MC (2007) Characterization of expressed sequence tags obtained by SSH during somatic embryogenesis in *Cichorium intybus* L. BMC Plant Biol. 7: 27–2229–7-27. 10.1186/1471-2229-7-27 PMC1913917 17553130

[77] Singla B, Tyagi AK, Khurana JP, Khurana P (2007) Analysis of expression profile of selected genes expressed during auxin-induced somatic embryogenesis in leaf base system of wheat (*Triticum aestivum*) and their possible interactions. Plant Mol Biol 65: 677–692. 17849219 10.1007/s11103-007-9234-z

[78] Vanneste S, Friml J (2009) Auxin: A trigger for change in plant development. Cell 136: 1005–1016. 19303845 10.1016/j.cell.2009.03.001

[79] Kieffer M, Neve J, Kępiński S (2010) Defining auxin response contexts in plant development. Curr Opin in Plant Biol 13: 12–20. 19942473 10.1016/j.pbi.2009.10.006

[80] Jenik PD, Barton MK (2005) Surge and destroy: the role of auxin in plant embryogenesis. Develop 132: 3577–3585. 10.1242/dev.01952 16077088

[81] Rademacher EH, Möller B, Lokerse AS, Llavata-Peris CI, van den Berg W, et al (2011) A cellular expression map of the Arabidopsis *AUXIN RESPONSE FACTOR* gene family. Plant J 68: 597–606. 21831209 10.1111/j.1365-313X.2011.04710.x

[82] Aida M, Vernoux T, Furutani M, Traas J, Tasaka M (2002) Roles of PIN-FORMED1 and MONOPTEROS in pattern formation of the apical region of the Arabidopsis embryo. Develop 129: 3965–3974. 10.1242/dev.129.17.3965 12163400

[83] Hadfi K, Speth V, Neuhaus G (1998) Auxin-induced developmental patterns in *Brassica juncea* embryos. Develop 125: 879–887. 10.1242/dev.125.5.879 9449670

[84] Choi YE, Katsumi M, Sano H (2001) Triiodobenzoic acid, an auxin polar transport inhibitor, suppresses somatic embryo formation and postembryonic shoot/root development in *Eleutherococcus senticosus* . Plant Sci 160: 1183–1190. 11337075 10.1016/s0168-9452(01)00357-0

[85] Larsson E, Sitbon F, Ljung K, von Arnold S (2008) Inhibited polar auxin transport results in aberrant embryo development in Norway spruce. New Phytol 177: 356–366. 18042199 10.1111/j.1469-8137.2007.02289.x

[86] Venkatesh K, Roja Rani A, Baburao N, Padmaja G (2009) Effect of auxins and auxin polar transport inhibitor (TIBA) on somatic embryogenesis in groundnut (*Arachis hypogaea* L.). African J Plant Sci 3: 288–293.

[87] Abrahamsson M, Valladares S, Larsson E, Clapham D, von Arnold S (2012) Patterning during somatic embryogenesis in Scots pine in relation to polar auxin transport and programmed cell death. Plant Cell Tiss Org Cult 109: 391–400.

[88] Tian C, Muto H, Higuchi K, Matamura T, Tatematsu K, et al (2004) Disruption and overexpression of auxin response factor 8 gene of Arabidopsis affect hypocotyl elongation and root growth habit, indicating its possible involvement in auxin homeostasis in light condition. Plant J 40: 333–343. 15469491 10.1111/j.1365-313X.2004.02220.x

[89] Mallory AC, Bartel DP, Bartel B (2005) MicroRNA-directed regulation of Arabidopsis AUXIN RESPONSE FACTOR17 is essential for proper development and modulates expression of early auxin response genes. Plant Cell 17: 1360–1375. 15829600 10.1105/tpc.105.031716 PMC1091760

[90] Ding Z, Friml J (2010) Auxin regulates distal stem cell differentiation in *Arabidopsis* roots. Proc Natl Acad Sci USA 107: 12046–12051. 20543136 10.1073/pnas.1000672107 PMC2900669

[91] Rensing SA, Lang D, Schumann E, Reski R, Hohe A (2005) EST sequencing from embryogenic *Cyclamen persicum* cell cultures identifies a high proportion of transcripts homologous to plant genes involved in somatic embryogenesis. J Plant Growth Regul 24: 102–115.

[92] Wu XM, Li FG, Zhang CJ, Liu CL, Zhang XY (2009) Differential gene expression of cotton cultivar CCRI24 during somatic embryogenesis. J Plant Physiol 166: 1275–1283. 19328593 10.1016/j.jplph.2009.01.012

[93] Thakare D, Tang W, Hill K, Perry SP (2008) The MADS-domain transcriptional regulator AGAMOUS-LIKE15 promotes somatic embryo development in Arabidopsis and soybean. Plant Physiol 146: 1663–1672. 18305206 10.1104/pp.108.115832 PMC2287341

[94] Che P, Lall S, Nettleton D, Howell SH (2006) Gene expression programs during shoot, root, and callus development in Arabidopsis tissue culture. Plant Physiol 141: 620–637. 16648215 10.1104/pp.106.081240 PMC1475446

[95] Lu J, Vahala J, Pappinen A (2011) Involvement of ethylene in somatic embryogenesis in Scots pine (*Pinus sylvestris* L.). Plant Cell Tiss Org Cult 107: 25–33.

[96] Zheng Q, Zheng Y, Perry SE (2013) AGAMOUS-Like15 promotes somatic embryogenesis in Arabidopsis and soybean in part by the control of ethylene biosynthesis and response. Plant Physiol 161: 2113–2127. 23457229 10.1104/pp.113.216275 PMC3613480

[97] Krishnaswamy S, Verma S, Rahman MH, Kav NNV (2011) Functional characterization of four APETALA2-family genes (*RAP2.6, RAP2.6L, DREB19* and *DREB26*) in Arabidopsis. Plant Mol Biol 75: 107–127. 21069430 10.1007/s11103-010-9711-7

[98] Asahina M, Azuma K, Pitaksaringkarn W, Yamazaki T, Mitsuda N, et al (2011) Spatially selective hormonal control of RAP2.6L and ANAC071 transcription factors involved in tissue reunion in Arabidopsis. Proc Natl Acad Sci USA 108: 16128–16132. 21911380 10.1073/pnas.1110443108 PMC3179063

[99] Werner T, Schmulling T (2009) Cytokinin action in plant development. Curr Opin Plant Biol 12: 527–538. 19740698 10.1016/j.pbi.2009.07.002

[100] Cheng X, Jiang H, Zhang J, Qian Y, Zhu S, et al (2010) Overexpression of type-A rice response regulators, OsRR3 and OsRR5, results in lower sensitivity to cytokinins. Genet Mol Res 9: 348–359. 20309821 10.4238/vol9-1gmr739

[101] Bishopp A, Benková E, Helariutta Y (2011) Sending mixed messages: auxin-cytokinin crosstalk in roots. Curr Opin Plant Biol 14: 10–16. 20926335 10.1016/j.pbi.2010.08.014

[102] Pernisova M, Klima P, Horak J, Valkova M, Malbeck J, et al (2009) Cytokinins modulate auxin-induced organogenesis in plants via regulation of the auxin efflux. Proc Natl Acad Sci USA 106: 3609–3614. 19211794 10.1073/pnas.0811539106 PMC2640219

[103] Rashotte AM, Mason MM, Hutchison CE, Ferreria FJ, Schaller GE, et al (2006) A subset of Arabidopsis AP2 transcription factors mediate cytokinin responses in concert with a two-component pathway. Proc Natl Acad Sci USA 103: 11081–11085. 16832061 10.1073/pnas.0602038103 PMC1544176

[104] Ishida K, Yamashino T, Yokoyama A, Mizuno T (2008) Three type-B response regulators, ARR1, ARR10 and ARR12, play essential but redundant roles in cytokinin signal transduction throughout the life cycle of *Arabidopsis thaliana* . Plant Cell Physiol 49: 47–57. 18037673 10.1093/pcp/pcm165

[105] Curaba J, Moritz T, Blervaque R, Parcy F, Raz V, et al (2004) *AtGA3ox2*, a key gene responsible for bioactive gibberellin biosynthesis, is regulated during embryogenesis by LEAFY COTYLEDON2 and FUSCA3 in Arabidopsis. Plant Physiol 136: 3660–3669. 15516508 10.1104/pp.104.047266 PMC527164

[106] Gaj MD, Trojanowska A, Ujczak A, Mędrek M, Kozioł A, et al (2006) Hormone-response mutants of *Arabidopsis thaliana* (L.) Heynh. impaired in somatic embryogenesis. Plant Growth Regul 49: 183–197.

[107] Jiang C, Fu X (2007) GA action: turning on de-DELLA repressing signalling. Curr Opin Plant Biol 10: 461–465. 17900970 10.1016/j.pbi.2007.08.011

[108] Achard P, Renou JP, Berthome R, Harberd NP, Genschik P (2008) Plant DELLAs restrain growth and promote survival of adversity by reducing the levels of reactive oxygen species. Curr Biol 18: 656–660. 18450450 10.1016/j.cub.2008.04.034

[109] Fridborg I, Kuusk S, Robertson M, Sundberg E (2001) The Arabidopsis protein SHI represses gibberellin responses in Arabidopsis and barley. Plant Physiol 127: 937–948. 11706176 PMC129265

[110] Zuo J, Niu Q, Chua N (2000) An estrogen receptor-based transactivator XVE mediates highly inducible gene expression in transgenic plants. Plant J 24: 265–273. 11069700 10.1046/j.1365-313x.2000.00868.x

[111] Kim SG, Lee AK, Yoon HK, Park CM (2008) A membrane-bound NAC transcription factor NTL8 regulates gibberellic acid-mediated salt signaling in Arabidopsis seed germination. Plant J 55: 77–88. 18363782 10.1111/j.1365-313X.2008.03493.x

[112] Olsson AS, Engström P, Söderman E (2004) The homeobox genes *ATHB-12* and *ATHB7* encode potential regulators of growth response to water deficit in Arabidopsis. Plant Mol Biol 55: 663–677. 15604708 10.1007/s11103-004-1581-4

[113] Thachter LF, Powell JJ, Aitken EAB, Kazan K, Manners JM (2012) The lateral organ boundaries transcription factor LBD20 functions in *Fusarium* wilt susceptibility and jasmonate signaling in Arabidopsis. Plant Physiol 60: 407–418. 10.1104/pp.112.199067 PMC3440215 22786889

[114] Denekamp MM, Smeekens SC (2003) Integration of wounding and osmotic stress signals determines the expression of the *MYB104* transcription gene. Annu Rev Plant Physiol 132: 1415–1423. 10.1104/pp.102.019273 PMC167081 12857823

[115] Krebs J (2009) Molecular and physiological characterization of DOF transcription factors in the model plant *Arabidopsis thaliana*. University of Potsdam, PhD thesis.

[116] Gaj MD (2011) Somatic embryogenesis and plant regeneration in the culture of *Arabidopsis thaliana* (L.) Heynh. immature zygotic embryos. In: Plant Embryo Culture. Methods in Molecular Biology. Thorpe TA and Yeung EC (eds), Humana Press, Totowa, New Jersey vol. 710: 257–265. 10.1007/978-1-61737-988-8_18 21207274

[117] Gamborg OL, Miller RA, Ojima K (1968) Nutrient requirements of suspension cultures of soybean root cells. Exp Cell Res 50: 151–158. 5650857 10.1016/0014-4827(68)90403-5

[118] Balazadeh S, Riaño-Pachón DM, Mueller-Roeber B (2008) Transcription factors regulating leaf senescence in *Arabidopsis thaliana.* . Plant Biol 1: 63–75. 10.1111/j.1438-8677.2008.00088.x 18721312

[119] Balazadeh S, Siddiqui H, Allu AD, Matallana-Ramirez LP, Caldana C, et al (2010) A gene regulatory network controlled by the NAC transcription factor ANAC092/AtNAC2/ORE1 during salt-promoted senescence. Plant J 62: 250–264. 20113437 10.1111/j.1365-313X.2010.04151.x

[120] R Core Team (2012) R: A language and environment for statistical computing. R Foundation for Statistical Computing, Vienna, Austria. URL: http://www.R-project.org/.

[121] Alexandrova KS, Conger BV (2002) Isolation of two somatic embryogenesis-related genes from orchardgrass (*Dactylis glomerata*). Plant Sci 162: 301–307.

