# Supplementary figures and images for: Extensive Modulation of the Transcription Factor Transcriptome during Somatic Embryogenesis in Arabidopsis thaliana
Source: PLoS One. 2013 Jul 17;8(7):e69261. doi: 10.1371/journal.pone.0069261 (PMC3714258; doi:10.1371/journal.pone.0069261)

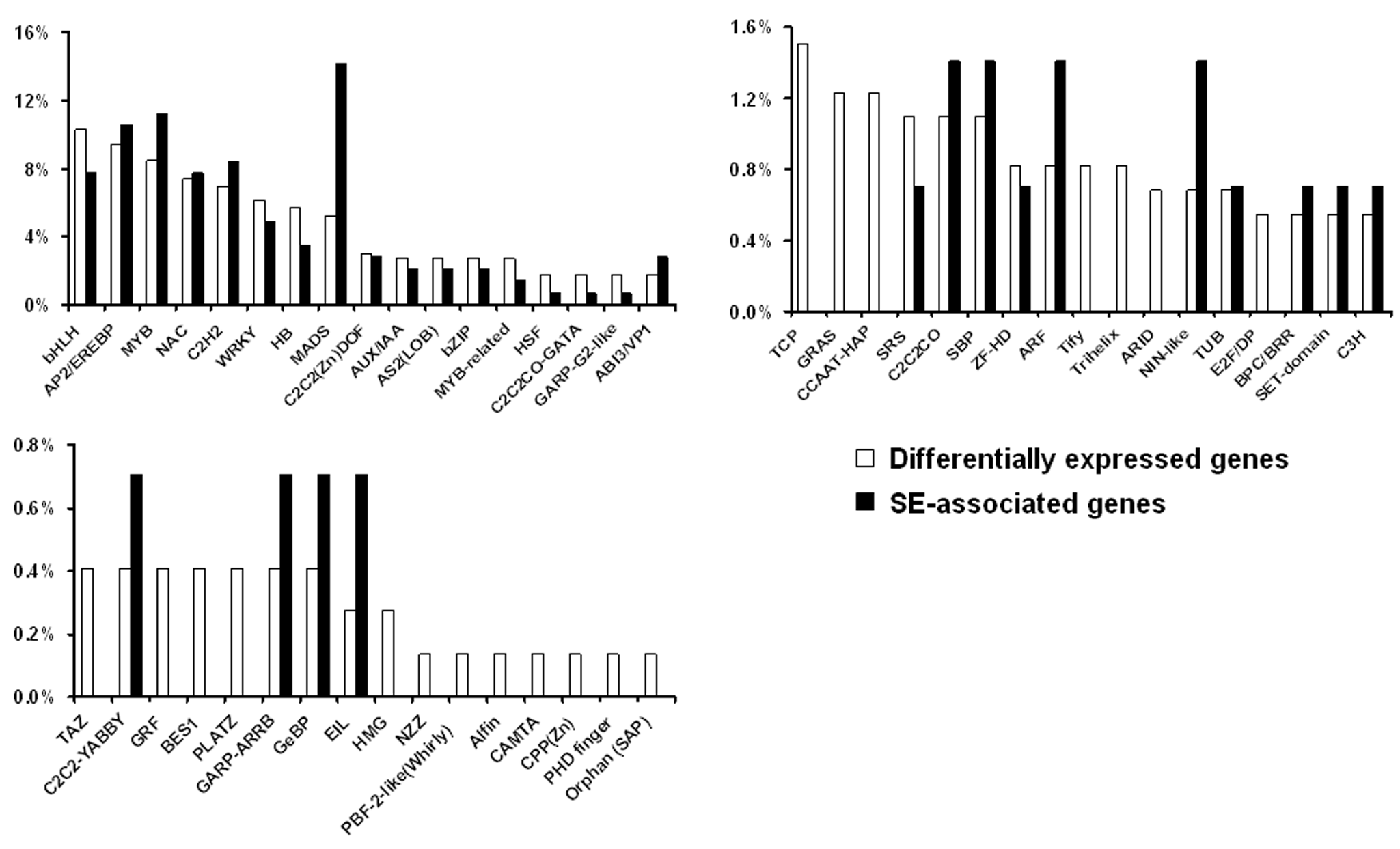

Supplement: Figure S1 — TF families among differentially expressed and SE-associated genes. For each TF family the percentage of genes differentially expressed or being SE-associated is indicated. (TIF) [file pone.0069261.s001.tif]

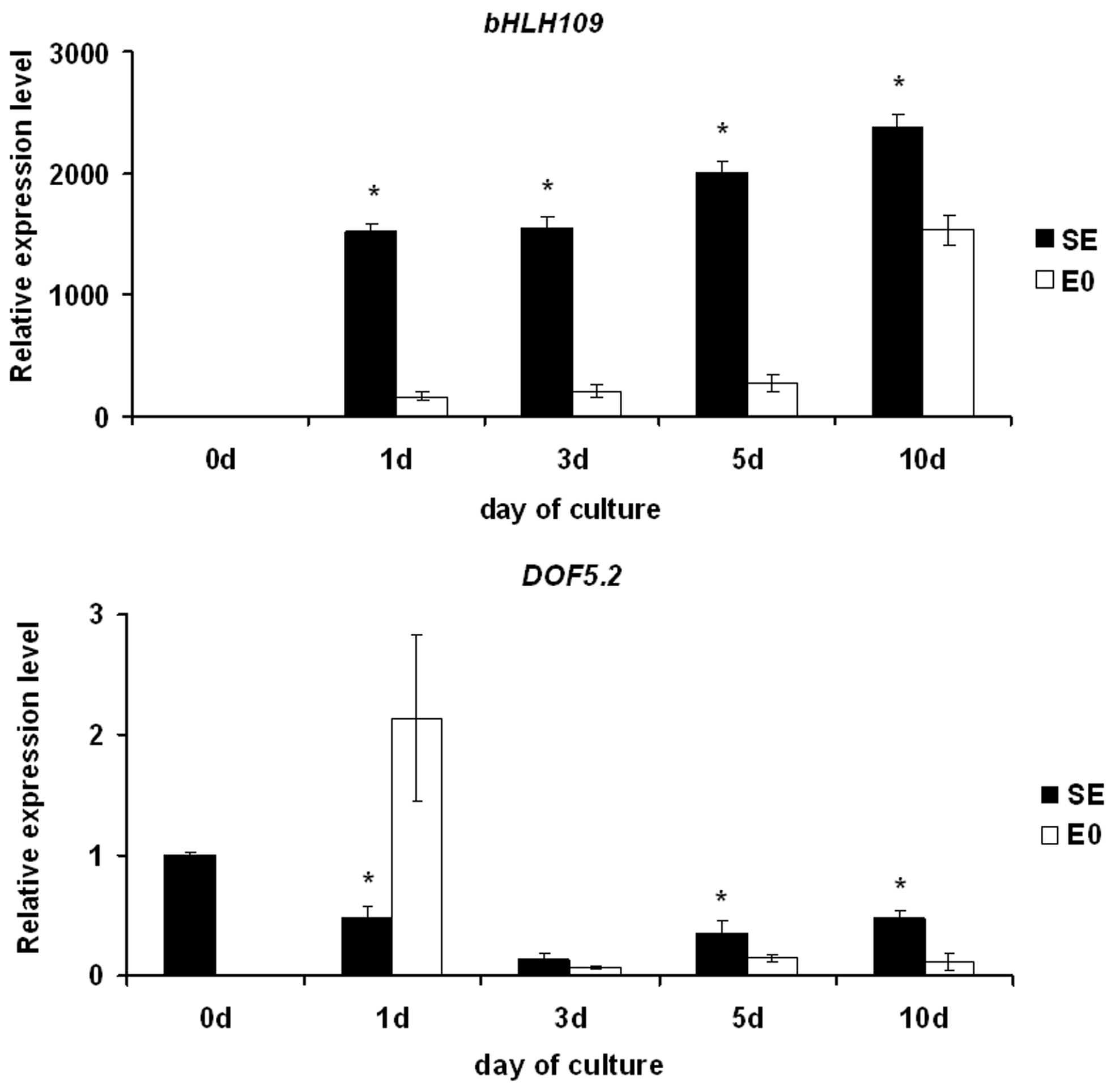

Supplement: Figure S2 — Expression levels of bHLH109 and DOF5.2 TFs in explants induced towards alternative morphogenic pathways, i.e. somatic embryogenesis (SE) and seedling development (E0). Values significantly different from E0 are marked by asterisks (n = 3; means ± SD are given; Mann-Whitney’s U test; p<0.05). (TIF) [file pone.0069261.s002.tif]
