# Supplementary material for: Extensive Modulation of the Transcription Factor Transcriptome during Somatic Embryogenesis in Arabidopsis thaliana
Source: PLoS One. 2013 Jul 17;8(7):e69261. doi: 10.1371/journal.pone.0069261 (PMC3714258; doi:10.1371/journal.pone.0069261)
Supplement: Table S1 — TFs exclusively or highly expressed in embryogenic Col-0 explants compared to non-embryogenic tanmei mutant explants. (DOC) [file pone.0069261.s003.doc]

**Table S1. TFs exclusively or highly expressed in embryogenic Col-0 explants compared to non-embryogenic *tanmei* mutant explants.**

Genes (41) exclusively expressed in Col-0 explants

| **AGI** | **Gene name** | **TF family** | **Known or predicted function** | **Expression level**  **(40-dCt)** | **Expression pattern**  **in SE*** |
| --- | --- | --- | --- | --- | --- |
| AT1G09250 | bHLH149/ EN144 | bHLH |  | 33.72 | Up-regulated in both SE stages |
| AT5G13330 | RAP2.6L | AP2/EREBP | Biotic stress, ethylene | 34.93 |
| AT3G23030 | IAA2 | Aux/IAA | Auxin | 34.45 |
| AT5G43700 | IAA4 | Aux/IAA | ZE [49] | 33.93 |
| AT3G44750 | ATHD2A | C2H2 | Histone deacetylase; reproductive development | 34.44 |
| AT2G22430 | ATHB6 | HB | Response to water deprivation | 34.14 |
| AT3G03450 | RGL2 | GRAS | Hyperosmotic salinity response;  negative regulator of the response to GA | 33.61 |
| AT4G36990 | HSFB1/HSF4 | HSF | Response to heat | 34.25 |
| AT5G47370 | HAT2 | NAC | Auxin-mediated morphogenesis | 32.25 |
| AT2G38470 | WRKY33 | WRKY | Defense response to fungus | 32.55 |
| AT1G71260 | ATWHY2 | PBF-2-like(Whirly) | Stress | 34.95 |
| AT5G09330 | ANAC082/VNI1 | NAC | Xylem vessel differentiation | 34.85 |
| AT5G14000 | ANAC084 | NAC | ZE [49] | 33.98 |
| AT1G25280 | TLP10 | TUB | Protein degradation | 34.84 |
| AT4G14720 | PPD2 | ZIM | Leaf development | 34.86 |
| AT4G24150 | GRF8 | GRF | Leaf development | 34.25 | Down-regulated in both SE stages |
| AT3G51910 | HSFA7A | HSF | Response to heat | 34.96 |
| AT1G25310 | MEE8 | bHLH | Seed/embryo development | 33.84 | Up during induction and down in advanced SE stage |
| AT2G36080 |  | ABI3/VP1 |  | 34.47 | Steady expression |
| AT1G36060 |  | AP2/EREBP |  | 34.35 |
| AT5G47220 |  | AP2/EREBP | JA | 33.63 |
| AT2G40220 | ABI4 | AP2/EREBP | Response to osmotic stress; ZE [45] | 34.81 |
| AT1G59750 | ARF1 | ARF | ZE [45] | 34.98 |
| AT2G47520 |  | AP2/EREBP |  | 34.67 |
| AT3G17609 |  | bZIP | Response to UV-B | 34.01 |
| AT5G01160 |  | C2H2 |  | 34.91 |
| AT4G37180 |  | GARP-G2-like |  | 34.94 |
| AT3G11260 | WOX5 | HB | QC; ZE [30]; cultured roots, somatic embryo;  SE in *Medigaco truncatula* [47] | 31.23 |
| AT3G46600 |  | GRAS |  | 34.88 |
| AT4G40060 |  | HB | Photoperiodism, flowering | 33.01 |
| AT5G23090 |  | CCAAT |  | 34.78 |
| AT5G19520 |  |  | Detection of mechanical stimulus | 34.24 |
| AT2G22300 |  | CAMTA | Response to biotic stimulus | 34.56 |
| AT3G24120 |  | GARP-G2-like |  | 3498 |
| AT3G10113 |  | MYB-related |  | 34.29 |
| AT1G06180 |  | MYB | Reponse to salt stress | 33.82 |
| AT5G67580 |  | MYB-related | Reponse to salt stress | 34.53 |
| AT1G52890 |  | NAC | Response to water deprivation | 34.26 |
| AT1G76880 |  | Trihelix |  | 34.63 |
| AT2G42400 |  | VOZ |  | 34.80 |

*Expression level in two subsequent SE stages, induction (5d - 0d) and advanced (10d - 5d) stages were considered

Genes (108) highly up-regulated in Col-0 vs. *tanmei* explants

| **AGI** | **Gene name** | **TF family** | **Known or predicted function** | **Fold change**  **Col-0 vs. *tan*** | **Expression pattern in SE* (Col-0)** |
| --- | --- | --- | --- | --- | --- |
| AT1G55650 |  | ARID |  | 252.48 | Up-regulated in both SE stages |
| AT5G58280 |  | ABI3/VP1 |  | 28.25 |
| AT3G27940 | LBD26 | AS2 (LOB) I |  | 16.00 |
| AT1G80580 |  | AP2/EREBP | ZE [49] | 12.30 |
| AT1G10480 | ZFP5 | C2H2 |  | 96.34 |
| AT4G20970 |  | bHLH | Defense response to fungus | 45.57 |
| AT5G06650 | GIS2 | C2H2 | GA, cytokinins | 57.28 |
| AT4G01260 |  | GeBP |  | 36.50 |
| AT1G68320 | MYB62 | MYB | Stress | 90.51 |
| AT4G19630 |  | HSF |  | 122.79 |
| AT5G27810 |  | MADS |  | 12.73 |
| AT2G21650 | MEE3 | MYB-related | Seed/embryo development | 46.53 |
| AT1G12260 | EMB2749/ANAC007 | NAC | Seed/embryo development | 13.45 |
| AT4G38340 |  | NIN-like | Flower development | 15.35 |
| AT3G04430 | ANAC049 | NAC |  | 12.47 |
| AT5G46590 |  | NAC | Shoot regeneration | 106.15 |
| AT1G29860 | WRKY71 | WRKY | Stress, ethylene | 11.63 |
| AT1G33760 | ERF022 | AP2/EREBP |  | 249.00 | Down-regulated in both SE stage |
| AT2G39250 | SNZ | AP2/EREBP | Flowering | 62.25 |
| AT2G31220 |  | bHLH |  | 49.18 |
| AT1G68190 |  | C2C2(Zn) CO-like |  | 1351.18 |
| AT2G22800 | HAT9 | HB |  | 11.47 |
| AT5G10120 |  | EIL | Seed/embryo development | 33.36 |
| AT4G11880 | AGL14 | MADS | Root development | 15500.21 |
| AT5G27130 | AGL39 | MADS | Flower development | 40.50 |
| AT5G62470 | MYB96 | MYB | Response to salt | 58.89 |
| AT2G30420 | ETC2 | MYB-related | Seed/embryo development | 12.73 |
| AT4G37610 | BT5 | TAZ | Leaf development | 11.39 |
| AT2G28500 | LBD11 | AS2 (LOB) I |  | 16.80 | Up during induction and down in advanced SE stage |
| AT1G69180 | CRC | C2C2(Zn) YABBY | Flower development | 142.02 | Down during induction and up in advanced SE stage |
| AT2G35640 |  | MYB-RELATED |  | 11.39 | Steady expression |
| AT5G45980 |  | HB |  | 205.07 |
| AT3G18550 |  | TCP |  | 100.43 |
| AT5G63900 |  | PHD finger | ZE [49] | 249.00 |
| AT1G56010 |  | NAC |  | 25.81 |
| AT3G59580 |  | RWP-RK |  | 98.36 |
| AT1G32510 |  | NAC |  | 195.36 |
| AT1G34180 |  | NAC |  | 195.06 |
| AT1G74480 |  | RWP-RK |  | 21.26 |
| AT2G03710 |  | MADS |  | 14.42 |
| AT4G00540 |  | MYB |  | 16.91 |
| AT1G48000 |  | MYB | Response to salt | 10.20 |
| AT1G49010 |  | MYB | Response to salt | 13.45 |
| AT2G42830 |  | MADS |  | 10.78 |
| AT4G25560 |  | MYB | ZE [49] | 16.91 |
| AT5G61620 |  | MYB-related |  | 27.10 |
| AT5G59340 |  | HB | Microsore embryogenesis in *Brassica napus* [53] | 20.39 |
| AT1G02230 |  | NAC |  | 91.77 |
| AT1G02250 |  | NAC |  | 172.45 |
| AT3G12820 |  | MYB |  | 53.08 |
| AT3G13890 |  | MYB | Secondary cell wall | 224.41 |
| AT1G22590 |  | MADS |  | 14.93 |
| AT1G31630 |  | MADS |  | 9.92 |
| AT1G54760 |  | MADS |  | 10.27 |
| AT1G65330 |  | MADS |  | 62.25 | Steady expression |
| AT5G39750 |  | MADS |  | 128.89 |
| AT3G61230 |  | LIM |  | 53.45 |
| AT5G61420 |  | MYB | Stress | 23.26 |
| AT5G62320 |  | MYB |  | 22.78 |
| AT2G26960 |  | MYB |  | 39.67 |
| AT2G31180 |  | MYB | Response to salt | 28.25 |
| AT5G35550 |  | MYB | ZE [49] | 22.01 |
| AT5G54070 |  | HSF |  | 156.50 |
| AT5G26630 | AGL35 | MADS | ZE [38] | 50.21 |
| AT5G07700 |  | MYB |  | 38.05 |
| AT5G11050 |  | MYB |  | 32.67 |
| AT2G06020 |  | G2-LIKE |  | 14.52 |
| AT3G13840 |  | GRAS |  | 11.16 |
| AT2G38300 |  | G2-LIKE |  | 38.05 |
| AT5G56780 |  | HRT |  | 41.64 |
| AT4G11250 |  | MADS |  | 91.14 |
| AT5G60470 |  | C2H2 |  | 12.30 |
| AT5G61470 |  | C2H2 |  | 11.16 |
| AT4G17810 |  | C2H2 |  | 13.45 |
| AT4G35280 |  | C2H2 |  | 29.65 |
| AT1G21970 |  | CCAAT |  | 18.00 |
| AT5G06800 |  | G2-LIKE |  | 4672.57 |
| AT1G68520 |  | C2C2(Zn) CO-like | SE in *Medigaco truncatula* [47] | 45.25 |
| AT4G21340 |  | BHLH |  | 48.50 |
| AT4G30180 |  | BHLH |  | 18.64 |
| AT2G18490 |  | C2H2 | ZE [38] | 10.70 |
| AT4G21050 |  | C2C2-DOF |  | 20.53 |
| AT2G37740 |  | C2H2 |  | 65.80 |
| AT5G06839 |  | bZIP |  | 70.52 |
| AT5G39860 |  | BHLH | ZE [49] | 40.22 |
| AT5G08141 |  | BZIP |  | 13.00 |
| AT5G15830 |  | BZIP |  | 115.36 |
| AT3G20750 |  | C2C2(Zn) GATA |  | 50.56 |
| AT3G49930 |  | C2H2 |  | 112.99 |
| AT1G25440 |  | C2C2-CO-LIKE |  | 22.16 |
| AT4G15250 |  | C2C2-CO-LIKE |  | 12.21 |
| AT1G26610 |  | C2H2 |  | 89.26 |
| AT2G17770 |  | BZIP |  | 21.86 |
| AT1G47650 |  | C2C2(Zn) DOF |  | 37.79 |
| AT1G26680 |  | ABI3/VP1 | ZE [49] | 10.13 |
| AT2G16210 |  | ABI3/VP1 |  | 20.97 |
| AT2G31215 |  | bHLH |  | 41.64 |
| AT2G01200 |  | AUX/IAA |  | 18.64 |
| AT5G35900 | LBD35 | AS2 (LOB) I | ZE [38] | 1509.65 |
| AT1G31050 |  | BHLH |  | 209.38 |
| AT1G49770 |  | bHLH |  | 27.86 |
| AT1G12540 |  | BHLH |  | 18.25 |
| AT1G76110 |  | ARID |  | 39.95 |
| AT1G06280 |  | AS2/LOB |  | 79.34 |
| AT4G31660 |  | ABI3/VP1 |  | 34.30 |
| AT1G36000 |  | AS2 (LOB) I |  | 266.87 |
| AT2G19510 |  | AS2(LOB) |  | 14.83 |
| AT5G57720 |  | ABI3/VP1 |  | 99.73 |
| AT2G23660 |  | AS2 (LOB) I |  | 2320.15 |
| AT4G00260 |  | ABI3/VP1 |  | 1082.39 |

*Expression level in two subsequent SE stages, induction (5d - 0d) and advanced (10d - 5d) stages were considered.
