# Supplementary material for: Extensive Modulation of the Transcription Factor Transcriptome during Somatic Embryogenesis in Arabidopsis thaliana
Source: PLoS One. 2013 Jul 17;8(7):e69261. doi: 10.1371/journal.pone.0069261 (PMC3714258; doi:10.1371/journal.pone.0069261)
Supplement: Table S3 — TFs showing an at least 10-fold expression change during early culture stages. (DOC) [file pone.0069261.s005.doc]

**Table S3. TFs showing an at least 10-fold expression change during early culture stages.**

A function was described for a gene when its involvement in a biological process/function was experimentally backed up as described in PubMed (www.ncbi.nlm.nih.gov/pubmed) or TAIR (http://arabidopsis.org/index.jsp).

| **AGI code** | **Gene** | **TF family** | **Fold change**  **5 d – 0 d**  **2^ΔΔCt^** | **Fold change**  **10 d – 5 d**  **2^ΔΔCt^** | **Function** |
| --- | --- | --- | --- | --- | --- |
| AT3G20840 | *PLT1* | AP2/EREBP | 29,4 | -2,09 | Development of the root stem cell niche; essential for quiescent center (QC) specification and stem cell activity |
| AT3G23230 | *ERF98* | AP2/EREBP | 34.5 | -2.9 | Plays an important role in regulating ascorbic acid biosynthesis |
| AT3G25730 | *EDF3,* | AP2/EREBP | 157.5 | -3.0 |  |
| AT5G18000 | *REM18/VDD* | B3 | 1184.4 | -2.2 | Role in female gametophyte development |
| AT2G42430 | *ASL18/LBD16* | AS2 (LOB) I | 67.1 | 2.2 |  |
| AT1G28160 | *ERF087* | AP2/EREBP | 10.1 | -14.7 |  |
| AT5G11190 | *SHN2* | AP2/EREBP | 136.2 | -2.5 | Regulating metabolism of lipid and/or cell wall components |
| AT5G13910 | *LEP* | AP2/EREBP | 34636.3 | -4.5 | Response to gibberellin stimulus |
| AT5G18560 | *PUCHI* | AP2/EREBP | 28924.4 | -2.2 | Control of cell division patterns during lateral root primordia development; involved in the determination of floral meristem identity |
| AT3G27940 | *LBD26* | AS2 (LOB) I | 128.8 | -2.6 |  |
| AT1G22490 |  | bHLH | 32.8 | -5.3 |  |
| AT3G58190 | *ASL16/ LBD29* | AS2 (LOB) I | 439.5 | -16.7 | Role in lateral root formation |
| AT1G74500 | *TMO7* | bHLH | 22.0 | -2.7 | Differentially expressed in torpedo stage between cotyledon vs. root [47] |
| AT3G23030 | *IAA2* | Aux/IAA | 14.6 | -3.4 | Response to auxin stimulus |
| AT5G25890 | *IAA28* | Aux/IAA | 10.0 | -3,1 | Response to auxin stimulus;  expressed during callus differentiation in *Oryza sativa*  lateral root formation |
| AT2G40200 |  | bHLH | 22.7 | -2.4 | Differentially expressed in torpedo stage between cotyledon vs. root [47] |
| AT3G17100 |  | bHLH | 15.2 | -2.3 |  |
| AT1G10480 | *ZFP5* | C2H2 | 18.5 | -3.4 |  |
| AT1G68640 | *PAN* | bZIP | 11.7 | -2.0 | Essential for AG activation in early flowers of short-day-grown plants |
| AT1G67030 | *ZFP6* | C2H2 | 89.2 | -2.0 |  |
| AT4G37850 |  | bHLH | 34.0 | -3.0 | Differentially expressed in torpedo stage between cotyledon vs. root [47] |
| AT4G34000 | *ABF3* | bZIP | 11.6 | -2.6 | Response to salt stress; response to ABA stimulus |
| AT5G48560 |  | bHLH | 34.0 | -2.5 |  |
| AT5G56960 |  | bHLH | 39.9 | -5.1 | Response to chitin |
| AT5G58010 | *LRL3* | bHLH | 13.6 | -2.4 | Regulates root hair development |
| AT5G06510 | *NF-YA10* | CCAAT-HAP2 | 24.5 | -2.2 |  |
| AT2G35940 | *BLH1* | HB | 48.8 | -2.0 | Polar nucleus fusion; response to continuous far red light stimulus by the high-irradiance response system; response to ABA stimulus |
| AT5G48890 | *LATE FLOWERING* | C2H2 | 13.5 | -3.0 | Controls the transition to flowering |
| AT1G13300 | *HRS1* | GARP-G2-like | 471.1 | -3.0 | Cellular response to phosphate starvation |
| AT3G19070 |  | GARP-G2-like | 22.0 | 2.2 |  |
| AT1G34650 | *HDG10* | HB | 25.6 | -2.7 |  |
| AT3G30260 | *AGL79* | MADS | 14.3 | -9.0 |  |
| AT2G41690 | *HSFB3* | HSF | 382.6 | -2.2 |  |
| AT4G37940 | *AGL21* | MADS | 37.2 | -2.8 | Highly expressed in roots |
| AT5G06500 | *AGL96* | MADS | 10.6 | -2.9 | Differentially expressed in torpedo stage between cotyledons and roots [47] |
| AT5G20240 | *PI* | MADS | 13.9 | -4.1 | Required for the specification of petal and stamen identities |
| AT5G23260 | *AGL32/TT16* | MADS | 20.5 | -5.4 | Plays a maternal role in fertilization and seed development |
| AT1G17310 |  | MADS | 198.0. | -3.5 |  |
| AT5G62165 | *AGL42* | MADS | 55.7 | -15.5 | Involved in floral transition |
| AT3G24310 | *MYB71* | MYB | 195.3 | -7.4 |  |
| AT3G27920 | *ATGL1* | MYB | 22.0 | -4.5 | Induction of trichome development |
| AT1G09540 | *MYB61* | MYB | 12.9 | -2.1 | Role in ABA-independent regulation of stomatal pore size |
| AT1G71692 | *XAL1/AGL12* | MADS | 31.5 | -2.5 | involved in root cell differentiation and flowering time |
| AT4G05100 | *MYB74* | MYB | 9.9 | -2.9 | response to salt stress; response to jasmonic acid stimulus |
| AT2G22630 | *AGL17* | MADS | 330.8 | -5.3 | Positive regulation of long-day photoperiodism, flowering |
| AT1G66230 | *MYB20* | MYB | 25.8 | -3.8 |  |
| AT5G56840 |  | MYB-related | 27.8 | -5.0 |  |
| AT1G75520 | *SRS5* | SRS | 41.9 | -3.3 |  |
| AT1G66600 | *ABO3/WRKY63* | WRKY | 32.4 | -2.7 | Responses to ABA and drought stress |
| AT5G66700 | *ATHB53* | HB | 30152.7 | -10.2 | AtHB53 is auxin-inducible and its induction is inhibited by cytokinin, especially in roots, therefore it may be involved in root development |
| AT1G68800 | *BRC2* | TCP | 100.4 | -2.1 |  |
| AT2G40750 | *WRKY54* | WRKY | 19.1 | -4.9 | Regulation of defense response |
| AT3G01970 | *WRKY45* | WRKY | 357.0 | -2.9 |  |
| AT2G46770 | *EMB2301/ANAC043* | NAC | 67.6 | -2.2 | Secondary cell wall biogenesis |
| AT3G04070 | *ANAC047* | NAC | 34.2 | -2.0 |  |
| AT5G01900 | *WRKY62* | WRKY | 1734.1 | -2.9 | Pathogene response |
| AT5G13080 | *WRKY75* | WRKY | 44.9 | -3.9 | Response to stress |
| AT5G28650 | *WRKY74* | WRKY | 15.0 | -2.4 |  |
| AT3G44350 | *ANAC061* | NAC | 15393.1 | -2.0 |  |
| AT1G74660 | *MIF1* | ZF-HD | 24.9 | -2.6 |  |
| AT5G41090 | *ANAC095* | NAC | 374.8 | -5.3 |  |
| AT1G29280 |  | WRKY | 68.5 | -2.0 |  |
