# Supplementary material for: Extensive Modulation of the Transcription Factor Transcriptome during Somatic Embryogenesis in Arabidopsis thaliana
Source: PLoS One. 2013 Jul 17;8(7):e69261. doi: 10.1371/journal.pone.0069261 (PMC3714258; doi:10.1371/journal.pone.0069261)
Supplement: Table S4 — T-DNA insertion lines used for the functional analysis of selected TFs. (DOC) [file pone.0069261.s006.doc]

**Table S4. T-DNA insertion lines used for the functional analysis of selected TFs.**

| **Stock number**  **SALK lines** | **AGI code** | **Gene name** | **Homozygous**  **line, source** | **Insertion site** | **Expression level** |
| --- | --- | --- | --- | --- | --- |
| N537369 | *AT3G49940* | *LBD38* | *DG** | *Promoter* | *reduced* |
| N555766 | *AT1G19040* | *-* | *DG** | *Exon* | *not detected* |
| N575764 | *AT3G57600* | *DREB2F* | *DG** | *Promoter* | *reduced* |
| N587226 | *AT2G27300* | *NTL8* | *DG** | *Promoter* | *not detected* |
| N588507 | *AT5G66990* | *-* | *DG** | *Promoter* | *reduced* |
| N612011 | *AT2G37120* | *-* | *DG** | *Promoter* | *reduced* |
| N614874 | *AT5G39660* | *DOF5.2* | *DG** | *Promoter* | *reduced* |
| N669955 | *AT4G24540* | *AGL24* | *NASC* | *Exon* | *reduced* |
| N859974 | *AT4G05100* | *AtMYB74* | *NASC* | *Exon* | *not detected* |
| N680157 | *AT3G03760* | *LBD20* | *NASC* | *Intron* | *reduced* |
| N591690 | *AT1G33760* | *ERF022* | *DG** | *Exon* | *not detected* |
| N652235 | *AT3G17600* | *IAA31* | *DG** | *Promoter* | *reduced* |
| N565384 | *AT3G62100* | *IAA30* | *DG** | *Intron* | *reduced* |
| N639276 | *AT4G32280* | *IAA29* | *DG** | *Promoter* | *reduced* |
| N557573 | *AT3G04730* | *IAA16* | *DG** | *Promoter* | *reduced* |
| N678029 | *AT1G06170* | *bHLH89* | *NASC* | *Exon* | *not detected* |
| N872755** | *AT3G61890* | *ATHB-12* | *NASC* | *Promoter* | *reduced* |

DG*: Homozygous plants were selected in T_3_ segregating populations in the Department of Genetics, University of Silesia, Katowice, Poland, by using gene- and insert-specific primers. The primers were designed following the instructions made on the SALK webpage (http://signal.salk.edu/tdnaprimers.2.html).**, SAIL line.
