# Supplementary material for: Extensive Modulation of the Transcription Factor Transcriptome during Somatic Embryogenesis in Arabidopsis thaliana
Source: PLoS One. 2013 Jul 17;8(7):e69261. doi: 10.1371/journal.pone.0069261 (PMC3714258; doi:10.1371/journal.pone.0069261)
Supplement: Table S5 — Primers used for the amplification of open reading frames. (DOC) [file pone.0069261.s007.doc]

**Table S5. Primers used for the amplification of open reading frames.**

| **Gene name (AGI code)** | **Primer** |
| --- | --- |
| *NTL8* (AT2G27300) | NTL8-IOE-fwd - ATGTCTAAAGAAGCTGAGATG |
|  | NTL8-IOE-rev - TTAGTTCCTAGCTATTAATAC |
| *ERF022* (AT1G33760) | ERF022-IOE-fwd - ATGGAAAACACTTACGTTGGC |
|  | ERF022-IOE-rev - TCAATTATTAGAATTCCATATG |
| *bHLH89* (AT1G06170) | bHLH89-IOE-fwd - ATGGGAGGAGGAGGCATGTTTG |
|  | bHLH89-IOE-rev - TTAATCACTAGATAACAGTG |
| *bHLH109* (AT1G68240) | bHLH109-IOE-fwd - ATGGAGAGAAACAACCGCAA |
|  | bHLH109-IOE-rev - TTAAGGTTGAGTTTGATTTG |
| *REM22* (AT3G46770) | REM22-IOE-fwd - ATGGAAGAGAACTCAAG |
|  | REM22-IOE-rev - TCAGGCCTTGATAATGTG |
| *AGL2* (AT5G15800) | AGL2-IOE-fwd - ATGGGAAGAGGAAGAGTAGAG |
|  | AGL2-IOE-rev - TCAGAGCATCCACCCCGGGATG |
| *WRKY31* (AT4G22070) | WRKY31-IOE-fwd - ATGTTCCGGTTTCCAGTGAGTC |
|  | WRKY31-IOE-rev - TTATTGCCTACTGTCATTGTTG |
| *DOF5.2* (AT5G39660) | DOF5.2-IOE-fwd - ATGGCTGATCCGGCGATTAAGC |
|  | DOF5.2-IOE-rev - CTATGAGCTCTCATGGAAGTTTG |
