# Supplementary material for: Extensive Modulation of the Transcription Factor Transcriptome during Somatic Embryogenesis in Arabidopsis thaliana
Source: PLoS One. 2013 Jul 17;8(7):e69261. doi: 10.1371/journal.pone.0069261 (PMC3714258; doi:10.1371/journal.pone.0069261)
Supplement: Table S6 — Expression level of transgenes in seedlings treated with ß-estradiol (5 µM) for 2 days. (DOC) [file pone.0069261.s008.doc]

**Table S6:** Expression level of transgenes in seedlings treated with ß-estradiol (5 µM) for 2 days.

| **Transgenic line** | **Gene** | **Expression level (ΔCt)***  **untreated seedlings** | **Expression level (ΔCt)***  **estradiol-treated seedlings** | **Fold change**  **2^-ΔΔCt^** |
| --- | --- | --- | --- | --- |
| 1-4-1 | NTL8-IOE | 14.32 | 10.42 | 15 |
| 2-1-25 | ERF022-IOE | 12.42 | 1.46 | 1991 |
| 14-2-18 | bHLH89-IOE | 19.56 | 9.74 | 903 |
| 18-1-23 | bHLH109-IOE | 19.53 | 16.83 | 6 |
| 3-1-3 | REM22-IOE | 13.55 | 10.56 | 7.5 |
| 9-4-1 | AGL2-IOE | 11.98 | 8.28 | 13 |
| 4-2-1 | WRKY31-IOE | 18.37 | 16.34 | 4 |
| 14-2-9 | DOF5.2-IOE | 10.82 | 1.49 | 643 |

*Expression level (ΔCt) shown as difference in expression level between the selected TF gene and the reference gene *AT4G27090* encoding 60S ribosomal protein L14.
